# Evaluation of Antiproliferative Palladium(II) Complexes of Synthetic Bisdemethoxycurcumin towards In Vitro Cytotoxicity and Molecular Docking on DNA Sequence

**DOI:** 10.3390/molecules26144369

**Published:** 2021-07-20

**Authors:** Natalia Miklášová, Peter Herich, Juan Carlos Dávila-Becerril, Joaquín Barroso-Flores, Eva Fischer-Fodor, Jindra Valentová, Janka Leskovská, Jozef Kožíšek, Peter Takáč, Ján Mojžiš

**Affiliations:** 1 Department of Chemical Theory of Drugs, Faculty of Pharmacy, Comenius University in Bratislava, Kalinčiakova 8, 83104 Bratislava, Slovakia; peter.herich@stuba.sk (P.H.); valentova@fpharm.uniba.sk (J.V.); leskovska5@uniba.sk (J.L.); 2 Department of Physical Chemistry, Faculty of Chemical and Food Technology, Slovak University of Technology, Radlinského 9, 81237 Bratislava, Slovakia; jozef.kozisek@stuba.sk; 3 Instituto de Química Universidad Nacional Autónoma de México Circuito Exterior s/n Ciudad Universitaria, 04510 Ciudad de México, Mexico; juadb06@gmail.com; 4 Centro Conjunto de Investigación en Química Sustentable UAEM-UNAM, Carretera Toluca- Atlacomulco Km 14.5, C.P. 50200 Toluca Estado de México, Mexico; 5 Tumor Biology Department, Institute of Oncology “Prof. Dr. Ion Chiricuță”, 400015 Cluj-Napoca, Romania; fischer.eva@iocn.ro; 6 Department of Pharmacology, Faculty of Medicine, Pavol Jozef Šafárik University, Trieda SNP 1, 04011 Košice, Slovakia; takacp3@gmail.com (P.T.); jan.mojzis@upjs.sk (J.M.); 7 Department of Pharmacology and Toxicology, University of Veterinary Medicine and Pharmacy, Komenského 73, 04181 Košice, Slovakia

**Keywords:** palladium(II) complexes, synthetic bisdemethoxycurcumin, cytotoxicity, DNA-binding, HSA binding, transcription factor NF-κB, DFT calculations

## Abstract

Metallodrugs form a large family of therapeutic agents against cancer, among which is cisplatin, a paradigmatic member. Therapeutic resistance and undesired side effects to Pt(II) related drugs, prompts research on different metal–ligand combinations with potentially enhanced biological activity. We present the synthesis and biological tests of novel palladium(II) complexes containing bisdemethoxycurcumin (BDMC) **1** and **2**. Complexes were fully characterized and their structures were determined by X-ray diffraction. Their biological activity was assessed for several selected human tumor cell lines: Jurkat (human leukaemic T-cell lymphoma), HCT-116 (human colorectal carcinoma), HeLa (human cervix epitheloid carcinoma), MCF-7 (human breast adenocarcinoma), MDA-MB-231 (human mammary gland adenocarcinoma), A549 (human alveolar adenocarcinoma), Caco-2 (human colorectal carcinoma), and for non-cancerous 3T3 cells (murine fibroblasts). The cytotoxicity of **1** is comparable to that of cisplatin, and superior to that of **2** in all cell lines. It is a correlation between IC_50_ values of **1** and **2** in the eight studied cell types, promising a potential use as anti-proliferative drugs. Moreover, for Jurkat cell line, complexes **1** and **2**, show an enhanced activity. DFT and docking calculations on the NF-κB protein, Human Serum Albumin (HSA), and DNA were performed for **1** and **2** to correlate with their biological activities.

## 1. Introduction

Cancer is still one of the main causes of death in the world, for which one of the most common treatment for oncological diseases is based on combined therapies which use platinum metallodrugs such as cisplatin [1,2,3], oxaliplatin [4,5], carboplatin [4], nedaplatin [6], and lobaplatin [7,8]. However, treatment failure and multidrug resistance [9] in some cases has urged investigations in developing new metallic compounds with improved pharmacokinetic and medicinal characteristics as therapeutic alternatives. The use of metallic complexes is not limited to therapy, but includes the role of imaging agents [10] or even as dual therapeutic–imaging agents, as proven by some vanadium [11,12] and rhenium complexes. Metal-based complexes with more enhanced anti-cancer activity than conventional platinum drugs include metals such as ruthenium [13,14,15,16] and gold(III) [17,18], which have even been effective on cisplatin-resistant cell lines [19], as well as silver(I) complexes, which have proven increased cytotoxicity and selectivity as compared to cisplatin [20]. Successful attempts for greater cytotoxicity and fewer unwanted side-effects of platinum drugs were also achieved with the use of palladium complexes [21,22,23] and nanomaterials [24,25]. Several studies already confirmed the fact that palladium complexes show promising in vivo and in vitro antitumor activity. They are able to internalize in the cells, to modify the secondary structure of the DNA, and to inhibit the cell growth selectively towards the cancer cell lines, comparable to platinum-based anticancer drugs [26]. However, it was concluded that the efficacy of metallodrugs generally depends not only on the metal, but also on the ligand units coordinated to the metallic center. Dinuclear cyclopalladated complexes containing two bioactive ligands in a single molecule were designed. Among these biological ligands, curcumin, naturally found in Turmeric root, has been successfully used [27,28]. Palladium(II) complexes exhibited great cytotoxicity on human prostate cancer cells (DU145, LnCaP, PC3) which confirmed the potential of such organometallic derivatives to inhibit the tumor cell growth and to initiate the apoptosis [28]. Our previous research was also focused on the synthesis of palladium(II) complexes containing curcuminoids, which display a significant antiproliferative activity against several different human cancer cell lines [29,30,31,32,33]. Synthesized palladium complexes induced early and late apoptotic processes in colorectal cancer cells DLD-1 and HT-29 [30,32]. Moreover, the treatment of lymphocytes with palladium complexes containing a curcumin derivative reveals an increase in the proportion of the T helper CD4 positive cell population, collateral with the decrease of T effector CD8 positive cells, and in the case of A2780 ovary cancer cells and HT-29 colon cancer cells, a significant cytotoxicity is detected [31]. Lastly, our research on palladium complexes containing the β-diketo moiety suggested that such compounds will not exert severe side effects as anticancer drugs, since they displayed a limited toxicity against normal, healthy cell populations such as colon epithelial cells [30], lymphocytes [31], and hepatocytes [33] in vitro.

Several literature studies are also dedicated to assessing the biological activity of bisdemethoxycurcumin, the third component of turmeric extract. Based on the presumption that this minor component of turmeric is more stable as the other two derivatives (curcumin and demethoxycurcumin), significant anti-cancer properties have been observed. Bisdemethoxycurcumin inhibits cell proliferation, metastasis, and tumor growth and induces apoptosis in tumor cells. Moreover, it generates ROS levels in breast cancer, lung cancer, gastric cancer, and ovarian cancer [34,35]. Indeed, researches focused on the capacity of migration and invasion in HeLa cells, via the inhibition of NF-*κ*B, MMP-2, and -9 signaling pathways, presumed the possibility of using the BDMC as a potential preventive agent against human cervical cancer metastasis [36]. Although the promising activities exhibited by bisdemethoxycurcumin, the poor solubility and consequently weak bioavailability of this natural compound are still significant issues for the medical applications of this original natural product [37,38]. Several approaches for improving the drug delivery and activity of BDMC have been adopted. Here, we mention the optimization of BDMC with nanoparticles [39], preparation of microspheres for delivery of BDMC to specific cellular targets [37], or creation of metal-based complexes [40] to enhance the bioavailability, physicochemical properties, stability, medicinal effects, and so forth. Particularly, palladium complexes containing curcuminoids and BDMC displayed an increased cytotoxicity towards several human adenocarcinomas (MCF-7, HeLa, and A549), proving in this way the significance of the coordination of free ligands to a metal center [41].

In this work, we have synthesized and tested the cytotoxic activities of two Pd(II) complexes containing the 1,7-bis(4-hydroxyphenyl)hepta-1,6-diene-3,5-dione (BDMC) ligand, herein referred to as **1** and **2,** which were derived as a continuation of our work on palladium(II) complexes previously reported. Biological tests were performed for complexes **1** and **2** against Jurkat (human leukaemic T-cell lymphoma), HCT-116 (human colorectal carcinoma), HeLa (human cervix epitheloid carcinoma), MCF-7 (human breast adenocarcinoma), MDA-MB-231 (human mammary gland adenocarcinoma), A549 (human alveolar adenocarcinoma), Caco-2 (human colorectal carcinoma), and for non-cancerous 3T3 cells (murine fibroblasts) cell lines. Computational modeling of these compounds and their interactions with human serum albumin (HSA), the transcription factor NF-κB and a short DNA sequence was carried out to assess their cytotoxic properties.

Recent studies on diverse pytochemicals, pointed also the capacity of curcumin to regulate the signaling pathways through the inhibition of NF-κB transcription factor [42,43,44]. Moreover, it was reported that curcumin can interact with both serum albumins in cytoplasm [45] and DNA in nucleus [46]. Therefore, we found it important to perform in silico analyses on these two potential binding sites of our compounds.

## 2. Results and Discussion

### 2.1. Preparation and Structural Characterization of Palladium(II) Complexes

**Scheme 1:**
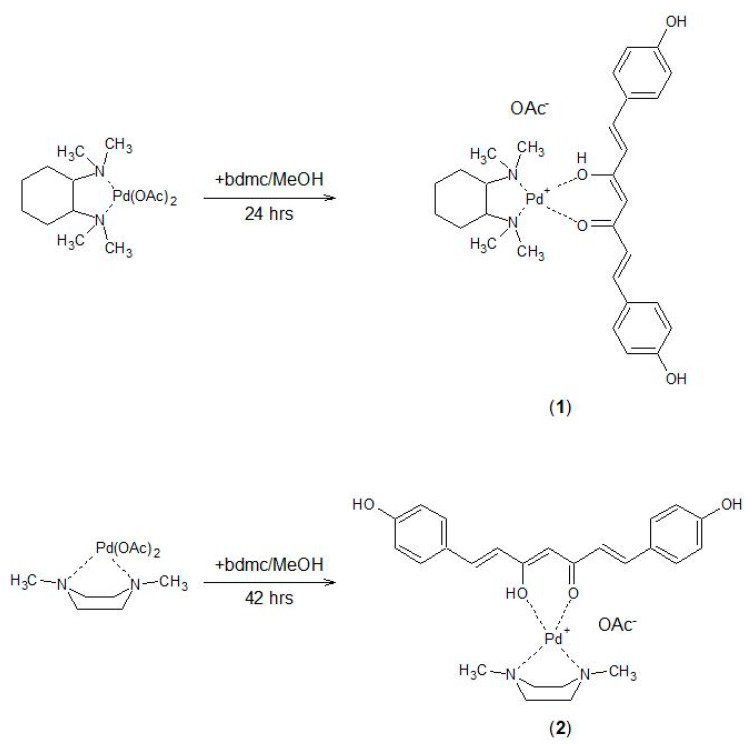
Synthetic pathways of palladium(II) complexes **1** and **2**.

Palladium(II) complexes **1** and **2** containing bisdemethoxycurcumin have been synthesized following a previously reported method [30,31,32,33] which consists of reacting the corresponding intermediate palladium(II) complexes with bisdemethoxycurcumin in an equimolecular ratio (Scheme 1). The two intermediate complexes were prepared from palladium(II) acetate and N,N,N′,N′-tetramethylcycohexane-1,2-diamine and N,N′-dimethylpiperazine, respectively [30]. Spectral methods (^1^H- and ^13^C-NMR, HR-MS and IR) confirmed the proposed structural formulas of compounds **1** and **2**. Moreover, complexes **1** and **2** were recrystallized from a mixture of methanol:acetonitrile (10:2) to form suitable crystals for X-ray diffraction.

**Figure 1:**
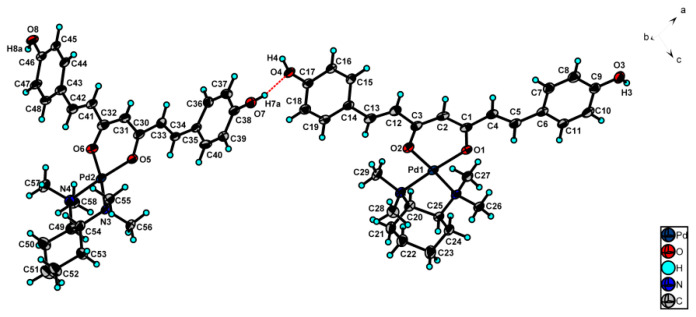
Arrangement of cations for complex **1**, including hydrogen interactions. Multi-disordered acetate anions and solvents molecules are omitted for clarity. Displacement ellipsoids are drawn at the 50% probability level.

**Figure 2:**
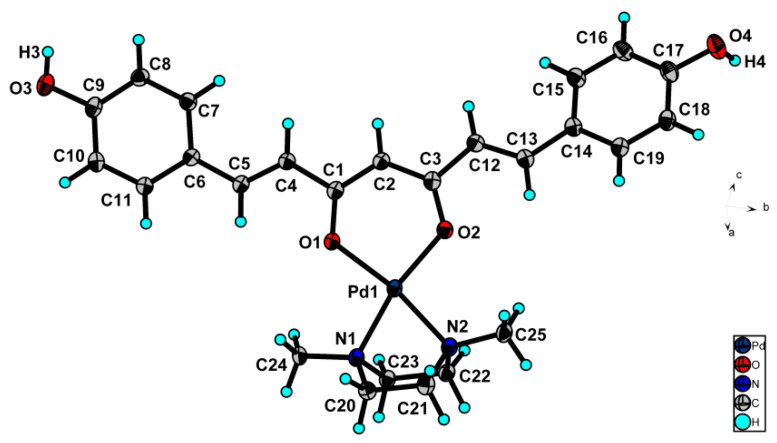
Arrangement of cations for complex **2**, including hydrogen interactions. Multi-disordered acetate anions and solvents molecules are omitted for clarity. Displacement ellipsoids are drawn at the 50% probability level.

The palladium(II) complex **1** crystallized in the C2 space group (No. 5), whereas complex **2** crystallized in the monoclinic P2_1_/n space group (No. 14). The molecular structures of the cationic part of complex **1** and **2** are shown in (Figure 1 and Figure 2) and their crystallographic data are summarized in (Table 1). 

The complex cations consist of bisdemethoxycurcumin ligand and appropriate amine ligand. Space disordered acetate anions and solvents were treated by OLEX2 software over the solvent mask (Supplementary Materials and CCDC cif files). The X- ray analyses of palladium(II) complexes **1** and **2** confirm that the disordered square-planar geometry around the central atoms is very close to the ideal square-planar arrangement of the coordination sphere. The polyhedron coordination of palladium atoms in complex **1** consists of two oxygen atoms of the carbonyl moieties of bisdemethoxycurcumin (first molecule: Pd1—O distances are 1.995(4) and 2.007(4) Å, while in the second molecule: Pd2—O distances are 1.982(4) and 2.002(4) Å) and two nitrogen atoms of N,N,N′,N′-tetramethylcycohexane-1,2-diamine (first molecule: Pd1—N distances are 2.042(5) and 2.057(4) Å, and in the second molecule Pd2—N distances are 2.039(5) and 2.051(4) Å). In the case of complex **2**, the polyhedron coordination of palladium atom contains two oxygen atoms of the carbonyl moieties of bisdemethoxycurcumin (Pd1—O distances are 1.991(2) and 1.987(2) Å) and two nitrogen atoms of N,N′-dimethylpiperazine (distances Pd1—N are 2.042(2) and 2.036(2) Å). A similarity between complex **2** and previously reported compounds [30] has been noticed. However, complex **1** shows slightly larger values for Pd-O and Pd-N lengths (Pd—O distances are 1.975(4) and 1.981(4) Å, and Pd—N are 2.038(5) and 2.045(5) Å). The angles around the central atom in complex **1** are in the range of 86.2(3)–94.7(2)° for both central atoms and 73.2(1)–96.41(9)° for complex **2**. The plane angles N1Pd1O2, N2Pd1O1, N3Pd2O6, and N4Pd2O5 are in the range of 173.5(1)–176.6(2)° for both central atoms in complex **1.** The plane angles N1Pd1O2 and N2Pd1O1 for complex **2** are in a range of 168.3(9)–169.6(9)° (Supplementary Table S1). Concerning the angles O-Pd-O and N-Pd-N, complex **1** shows a close similarity with the formerly reported compounds [30], while in complex **2** is observed a different value for the N1—Pd1—N2 angle (73.2°), which explains the better rigidity geometry of N,N′-dimethylpiperazine ligand. The crystal structures of complexes **1** and **2** are stabilized by a network of intramolecular and intermolecular hydrogen bonds and Van der Waals interactions. A zig-zag 3D network with big cavities containing multi-disordered solvent molecules (acetonitrile) and acetate anions is observed in both complexes **1** and **2** (Supplementary Table S2, Figures S3 and S4). The crystal structures (packing) of complexes **1** and **2** are given in Supplementary Figures S1 and S2. 

### 2.2. DFT Calculations

**Figure 3:**
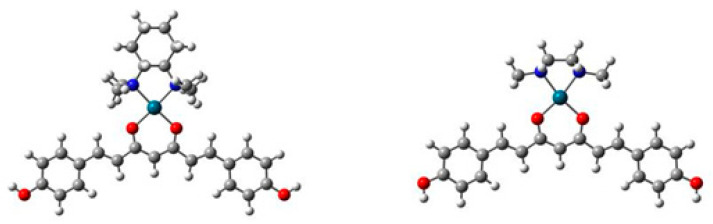
Optimized geometry of compounds **1** (**left**) and **2** (**right**) at the ωB97XD/LANL2DZ level of theory.

Geometry optimizations were performed starting from the crystallographic coordinates of compounds **1** and **2** at the ωB97XD/LANL2DZ level of theory with the use of the SMD continuous solvation model (water). The resulting structures (see Figure 3) were characterized as minima on the potential energy surface by means of vibrational calculations (Supplementary Table S3).

Energetic analysis of the Pd bonds was performed with the NBO3.1 code as provided by Gaussian 16. The sum of all Pd bond energies is 330.64 kcal/mol for compound **1** whereas for compound **2** it is 353.28 kcal/mol. The calculated bond energies in compound **1** are 115.28 kcal/mol for Pd-O and 73.09 kcal/mol for Pd-N bonds. For palladium complex **2**, the calculated bond energies are 127.45 kcal/mol for Pd-O and 74.67 kcal/mol for Pd-N bonds.

### 2.3. Docking Calculations

All docking simulations were performed with AutoDock Vina using an exhaustiveness factor of 10 since the binding energies dropped severely for subsequent binding modes making it futile to assess those conformations. Binding energies (−ΔG_b_) were obtained directly from the scoring functions set as default in Autodock Vina. 

#### 2.3.1. Human Serum Albumin (HSA)

**Figure 4:**
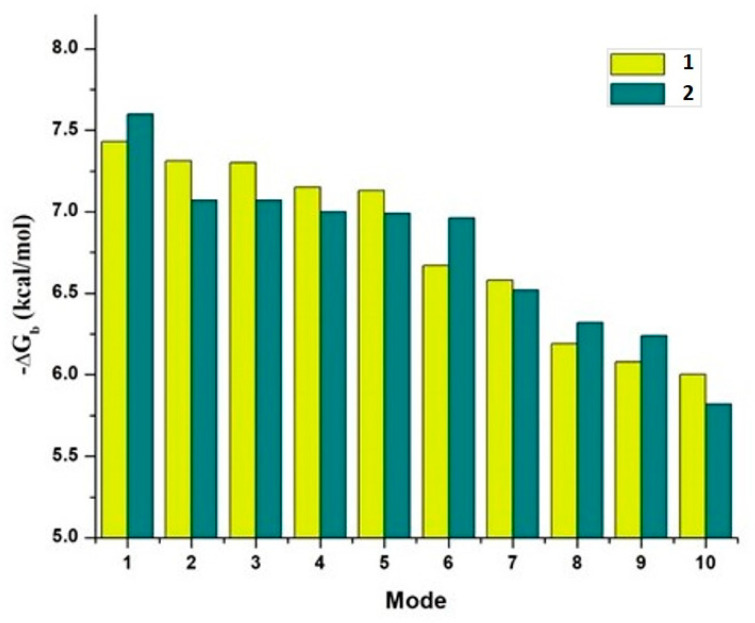
Binding energies [−kcal/mol] −ΔG_b_ for the ten highest-ranked binding modes for compounds **1** (yellow) and **2** (green) to HSA.

Both compounds **1** and **2** show good affinities for HSA (PDB entry 4F5S), as shown in Figure 4, where it can be observed that the Gibbs free energy of binding (−ΔG_b_) ranges between 6.00 and 7.43 kcal/mol for compound **1** and 5.82 to 7.60 kcal/mol for compound **2** for the 10 highest ranked modes of binding (Supplementary Table S4).

**Figure 5:**
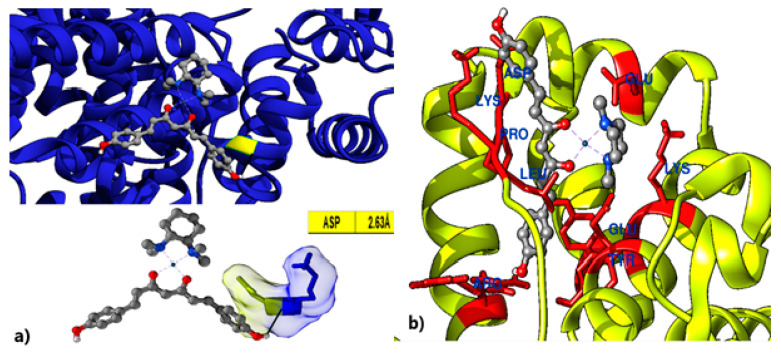
(**a**) Intermolecular interactions for compound **1** and HSA (**b**) Intermolecular interactions for compound **2** and HSA.

Figure 5a shows the intermolecular interactions for the complex HSA-**1** at the highest-ranked binding mode for which the strongest interaction is observed with a single asparagine amino acid at 2.6 Å, whereas compound **2** (Figure 5b) could only dock to an allosteric site in which it exhibits multiple interactions with amino acids Leu115, Lys116, Pro117, Asp118, Glu125, Lys136, Tyr137, Glu140, Tyr160, and Arg185.

#### 2.3.2. Transcription Factor NF-κB

**Figure 6:**
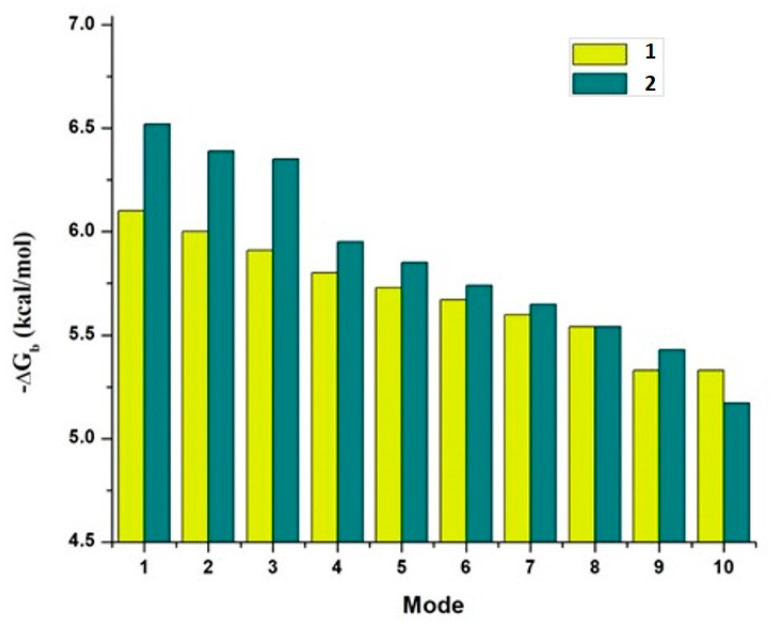
Binding energies [-kcal/mol] −ΔG_b_ for the ten highest ranked binding modes for compounds **1** (yellow) and **2** (green) to the transcription factor NF-κB.

Compound **2** exhibits a systematically higher affinity for NF-κB (PDB entry 1LE5) than compound **1,** as can be seen in Figure 6, where −ΔG_b_ ranges from 5.33 to 6.10 kcal/mol for compound **1** and from 5.17 to 6.52 kcal/mol for compound **2**. 

Although the difference in binding free energy is only very slight, on most of the highest ranked binding modes, NF-κB shows a higher preference for compound **2** which forms a stable hydrogen bond with ASP243 throughout all the ten highest binding modes (Supplementary Table S4).

#### 2.3.3. DNA Sequence

**Figure 7:**
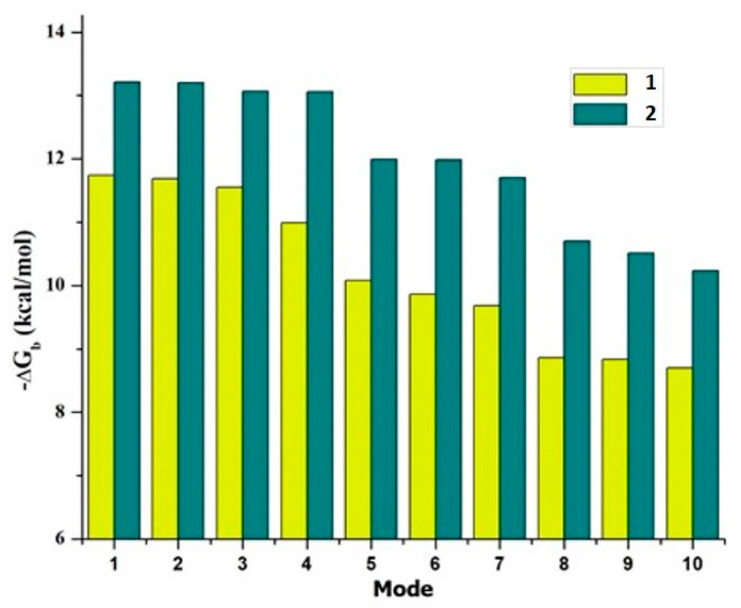
Binding energies −ΔG_b_ [-kcal/mol] for the ten highest ranked binding modes for compounds **1** (yellow) and **2** (green) to a DNA strand.

Both compounds **1** and **2** show a remarkable affinity for the minor grove of the selected DNA (PDB entry 2GVR) sequence, but the selectivity for compound **2** is much higher across the ten highest ranked binding modes (see Figure 7). −ΔG_b_ values for compound **1** range from 8.70 to 11.74 kcal/mol whereas for compound **2** they range from 10.23 to 13.21 kcal/mol.

**Figure 8:**
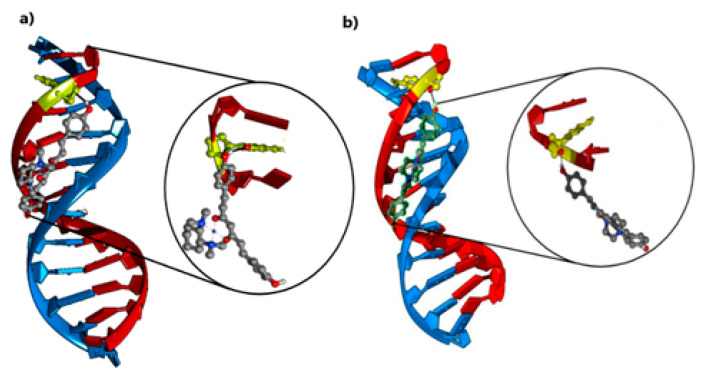
(**a**) DNA-**1** complex the inset shows a detail of complex **1** stacking to cytosine 11 with a mean interplanar distance of 2.46 Å (**b**) DNA-**2** complex the inset shows a detail of complex **2** stacking to cytosine 11 with a mean interplanar distance of 2.22 Å.

Although both compounds could be potential binders for DNA with various applications, compound **2** is much more likely to form a more stable association to DNA. In both cases, cytosine 11 is the base on which most of the binding modes rely for their interaction (see Figure 8 and Supplementary Table S4).

### 2.4. Biological Tests

**Figure 9:**
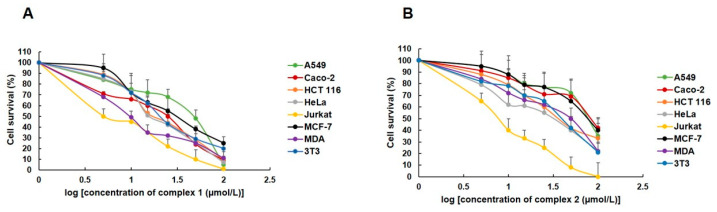
Sigmoidal dose-response curves for complex **1** (**A**) and **2** (**B**) in different cell lines after 72 h of incubation (data are presented as the mean + SD of three independent measurements performed in triplicate).

The newly synthesized palladium(II) complexes **1** and **2** were biologically tested on a series of human tumor cells: Jurkat, HCT 116, HeLa, MCF-7, MDA-MB-231, A549, Caco-2, and noncancerous murine fibroblasts 3T3. Mentioned cells were exposed to complexes **1** and **2** at different concentrations, from 5 to 100 µmol/L (Figure 9) and their cytotoxicity was compared with the effect of cisplatin on cell viability (Table 2, Supplementary Figure S5).

**Figure 10:**
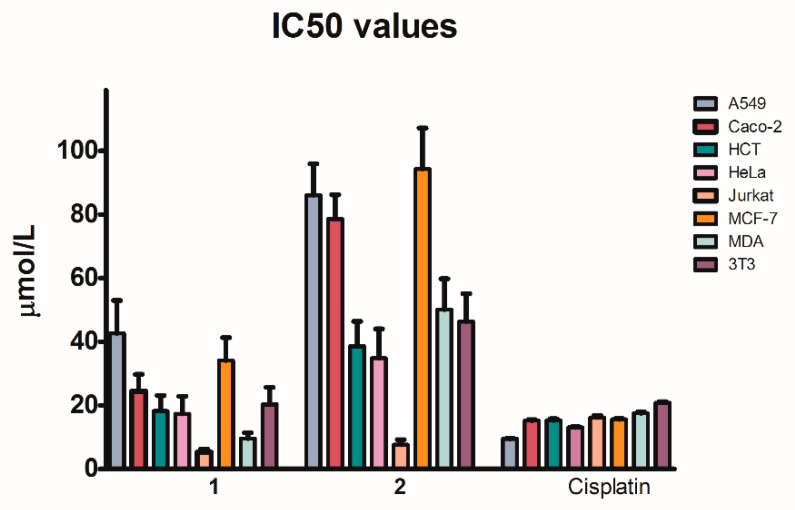
Cytotoxicity (IC_50_) of palladium(II) complexes 1, 2 and cisplatin (as standard) in 8 different cell lines.

Based on IC_50_ values, palladium(II) complex **1** shows overall better cytotoxicity than complex **2** (one-way analysis of variance and Bonferroni’s multiple comparison test in the 95% confidence interval, *p* < 0.05), displaying lower IC_50_ values in all cell lines (Table 2, Supplementary Figure S5). In A549, Caco-2, HCT, HeLa and MCF-7, complex **1** displayed higher IC_50_ values than cisplatin, thus being less active towards these five cell lines, while in Jurkat and MDA lines, complex **1** has a lower IC_50_ value than cisplatin, being more active. Complex **2** exhibits an inferior inhibitory effect to cisplatin among all cell lines, with the remarkable exception of Jurkat. Complex **2** has a higher IC_50_ value against the noncancerous fibroblasts 3T3 than cisplatin, whereas complex **1** exhibits a similar cytotoxicity as that of cisplatin. A good statistical correlation between the IC_50_ values of **1** and **2** through all the eight cell lines is observed (Spearman r 0.833, *p* value 0.015), which denotes that cytotoxicity caused by **1** and **2** tends to decrease in the same cell lines, notably for Jurkat, HeLa and HCT (Figure 10 and Supplementary Figure S5); no association was confirmed between the toxicities of cisplatin and the Pd(II) complexes **1** and **2** (Spearman correlation, *p* value 0.268 and *p* = 0.665, respectively). 

## 3. Materials and Methods

Chemicals used in syntheses (4-hydroxybenzaldehyde 99%, acetylacetone, B_2_O_3_, tri-*n*-butyl borate 98%, *n*-butanamine, ethylacetate, palladium(II) acetate, methanol, chloroform) were of reagent grade and were used as purchased. Bisdemethoxycurcumin was synthesized as described previously in the literature [47]. Intermediate palladium(II) complexes containing *N*,*N*,*N*′,*N*′-tetramethylcycohexane-1,2-diamine and *N*,*N*′-dimethylpiperazine, were prepared based on a reported [30].

For measurements of NMR spectra, a Varian Gemini 2000 spectrometer was used at a frequency of 300 MHz (for ^1^H-NMR) and 75 MHz (for ^13^C-NMR). All spectra were measured in CD_3_OD and the chemical shifts are reported relative to TMS used as an internal standard (Supplementary Materials).

The MS measurements were performed on an LTQ Orbitrap XL spectrometer using the electrospray ionization in positive mode. The operating parameters used: spray voltage (SV) 3.6 kV, sheath gas (Sh.G) 5 psi, capillary voltage (CV) 41 V, capillary temperature 275 °C. Spectra were recorded for both complexes (1 and 2) from *m*/*z* 150 to 800 (Supplementary Materials).

Infrared spectra were measured with a Nicolet 6700 FT-IR spectrophotometer in the range of 600–4000 cm^−1^ (Supplementary Materials).

### 3.1. Synthesis of Palladium(II) Complexes

Complex **1**: To a solution of 0.21 g (0.68 mmol) bisdemethoxycurcumin in methanol (7 mL) was added dropwise the methanolic solution (10 mL) of 0.27 g (0.68 mmol) intermediate palladium(II) complex containing *N,N,N′,N′*-tetramethylcycohexane-1,2-diamine. The reaction mixture turned from dark-orange to yellow and an orange precipitate was formed. The precipitate was filtered off from the mother liquor, dried and identified as the final product. Complex **1**: mp 184 °C (decomposed); 0.21 g yield (48%); ^1^H-NMR (CD_3_OD, 300 MHz), δ (ppm): 7.42–7.49 (m,^3^*J* = 8.6 Hz, 6H), 6.80 (d, ^3^*J* = 8.6 Hz, 4H), 6.65 (d, ^3^*J* = 15.7 Hz, 2H), 5.82 (s, 1H), 3.21 (d, 2H), 2.85 (s, 6H), 2.83 (s, 6H), 2.19 (d, 2H), 1.90 (s, 3H *OCOCH*_3_), 1.81 (2, 2H), 1.43–1.53 (m, 2 H), 1.21–1.29 (m, 2H). ^13^C-NMR (CD_3_OD, 75 MHz), δ (ppm): 178.6 (2C), 159.8 (2C), 140.3 (2C), 129.6 (4C), 126.5 (2C), 121.4 (2C), 115.5 (4C), 104.0 (1C), 71.7 (2C), 47.6 (2C), 42.1 (2C), 24.08 (2C), 23.82 (2C). IR υ (cm^−1^): 3010, 2943, 2867, 2675, 1599, 1497, 1437, 1389, 1273, 1162, 1103, 996, 970, 936, 829, 783, 703, 662, 633. HR-MS C_29_H_38_N_2_O_4_Pd^+^ calc.583.1783; exp. 583.1794.

Complex **2**: Bisdemethoxycurcumin (0.23 g; 0.73 mmol) was dissolved in 9 mL of methanol. To this solution was slowly added a solution of intermediate palladium(II) complex bounding *N,N′*-dimethylpiperazine (0.25 g; 0.73 mmol) in 8 mL of methanol. The reaction was checked on TLC after 24 h and unreacted bisdemethoxycurcumin it was observed, therefore the reaction was kept on stirring at room temperature another 18 h. After that, the solvent was removed and the final product was isolated from the mixture by silica gel chromatography, using as eluent methanol: chloroform in a ratio 1:9. Palladium complex 2 was obtained as an orange powder. Complex 2: mp 195 °C (decomposed); 0.20 g yield (46%); ^1^H-NMR (CD_3_OD, 300 MHz), δ (ppm): 7.50–7.45 (m, ^3^*J* = 8.6 Hz, 6H), 6.80 (d, ^3^*J* = 8.6 Hz, 4H), 6.65 (d, ^3^*J* = 15.7 Hz, 2H), 5.82 (s, 1H), 3.89 (d, *J* = 6.8 Hz, 4H), 2.74 (d, *J* = 6.8 Hz, 4H), 2.62 (s, 6H), 1.89 (s, 3H *OCOCH_3_*). ^13^C-NMR (CD_3_OD, 75 MHz), δ (ppm): 178.4 (2C), 159.7 (2C), 140.5 (2C), 129.6 (4C), 126.6 (2C), 121.2 (2C), 115.5 (4C), 104.3 (1C), 58.1 (4C), 45.2 (2C), 22.5 (1C). IR υ (cm^−1^): 3010, 2810, 2681, 2609, 1601, 1501, 1446, 1389, 1271, 1199, 1164, 1104, 994, 964, 828, 796, 704, 654. HR-MS C_25_H_30_N_2_O_4_Pd^+^ calc. 527.1157; exp. 527.1167.

### 3.2. X-ray Crystallography

Diffraction measurements were performed with a Stoe STADIVARI diffractometer equipped with Dectris Pilatus 300 K detector using a Genix3D Cu HF source (Cu-Kα, λ = 1.54186 Å) at 100 K employing a nitrogen gas open-flow cooler Cobra Oxford Cryosystems. Data reduction was achieved using X-Area (Stoe, 2018) software package [48]. The crystal structures of 1 and 2 were solved in OLEX2 software [49] using SHELXT-2015 program via Intrinsic Phasing [50] and refined with SHELXL-2015 by least-squares procedure on F^2^ [51]. All non-hydrogen atoms were refined with anisotropic thermal parameters. The positions of all hydrogen atoms in complexes 1 and 2, were geometrically optimized and constrained on their parent atoms. Thus, the constrained C—H bond lengths are: 0.95 Å (aromatic); 0.99 Å (aliphatic); 1.00 Å (asymmetric aliphatic); 0.98 Å (methyl group); and O—H bond length 0.84 Å (hydroxyl group) for complex 1. In the case of palladium complex 2, the C—H bond lengths are 0.93 Å (aromatic); 0.97 Å (aliphatic); 0.96 Å (methyl group) and the O—H length in hydroxyl group is 0.82 Å. The temperature factors of hydrogen were Uiso(H) = 1.2 Ueq(C) (for aromatic and aliphatic parts) and Uiso(H) = 1.5 Ueq(O, C) (for methyl and hydroxyl group). The DIAMOND (version 2.1e) [52], Mercury (version 4.1.3) [53] and OLEX2 (version 2-1.2) software [49] were used for the molecular graphics. The crystal structure of complex 1 contains eight-strong disordered anions per cell, which are removed, and electron density is calculated using OLEX2 solvent-masking. For complex 2, the cell contains one big cavity with four strong disordered acetate anions and eight acetonitrile solvent molecules. Solvent molecules and anions were removed and electron density was calculated as in the case of complex 1. Crystal data for palladium complexes 1 and 2, data collection procedures, structure determination, and refinement parameters are summarized in Table 1.

CCDC: 2044620-2044621 contains the supplementary crystallographic data for this paper. These data can be obtained free of charge via www.ccdc.cam.ac.uk/conts/retrieving.html (16 November 2020) (or from the CCDC, 12 Union Road, Cambridge CB2 1EZ, UK; Fax: +44-1223-336033; E-mail: deposit@ccdc.cam.ac.uk).

### 3.3. Computational Details

The electronic structure of compounds **1** and **2** was calculated with density functional theory-based methods at the ωB97XD/LANL2DZ level of theory with the Gaussian 16 suite of programs [54]. The LANL2DZ pseudopotential includes relativistic corrections to the core electrons which are important in describing Pd containing molecules. Molecular Docking computations on the NF-κB protein (PDB: 1LE5), human serum albumin (HSA, PDB: 4F5S) and a short DNA sequence (PDB: 2GVR) were performed for compounds **1** and **2** using the AutoDock 4.2.6 suite of programs [55]. This DNA sequence corresponds to a berenil-D(CGCGAATTCGCG)2 complex from which the rod-shaped aromatic ligand—similar in that regard to the BDMC ligand under study—in the major grove was removed, thus yielding a pre-docked DNA structure into which we could dock compounds **1** and **2**.

### 3.4. Biological Testing

The proliferation of cells was assessed from the absorbance at 490 nm wavelength with an automated Cytation™ 3 Cell Imaging Multi-Mode Reader (Biotek, Winooski, VT, USA).

Cell cultures: Seven human cancer cell lines and one noncancerous cell line were used for testing the synthesized complexes **1** and **2**. The Jurkat (human leukaemic T-cell lymphoma), HCT 116 (human colorectal carcinoma) and HeLa (human epitheloid cervix carcinoma) lines were cultured in RPMI 1640 medium (Biosera, Kansas City, MO, USA). The MCF-7 (human breast adenocarcinoma), MDA-MB-231 (human mammary gland adenocarcinoma), A549 (human alveolar adenocarcinoma), Caco-2 (human colorectal carcinoma), and noncancerous 3T3 (murine fibroblasts) cell lines were maintained in a growth medium consisting of high glucose Dulbecco’s Modified Eagle Medium with sodium pyruvate (GE, Healthcare, Piscataway, NJ, USA).

Cell cultivation: The growth medium was supplemented with a 10% fetal bovine serum, 1X HyClone™ Antibiotic/Antimycotic Solution (GE Healthcare, Little Chalfont, UK). Cells were cultured in an atmosphere containing 5% CO_2_ in humidified air at 37 °C. Cell viability, estimated by trypan exclusion, was greater than 95% before each experiment.

MTS cell proliferation/viability assay: The cytotoxicity of complexes 1 and 2 was determined by MTS (3-(4,5-dimethylthiazol-2-yl)-5-(3-carboxymethoxyphenyl) -2-(4-sulfophenyl)-2H-tetrazolium) assay. Cells were seeded at a density of 5 × 10^3^/well in 96-well polystyrene microplates (SARSTEDT, Nümbrecht, Germany). After 24 h, tested compounds **1** and **2** were added in various concentrations (100, 50, 10, and 5 μmol/L). After 72 h of incubation, 10 µL of MTS (5 mg/mL, Sigma-Aldrich Chemie, Steinheim, Germany) was added to each well according to the CellTiter 96^®^ Aqueous One Solution Cell Proliferation Assay protocol. The absorbance was measured at 490 nm with the automated Cytation™ 3 Cell Imaging Multi-Mode Reader (Biotek, Winooski, VT, USA) after 1 h of incubation. Three independent experiments were performed for each test. The results obtained from the MTS assay were used to determine the half-maximal inhibitory concentration (IC_50_) of each tested compound.

## 4. Conclusions

The NF-κB transcription factor is responsible for the fine tuning of the apoptotic process, cell cycle regulation, and cell differentiation in tumors. The crystal structure of NF-κB has two distinct druggable areas: the protein–protein interaction and the DNA-binding region [56]. The computational analysis showed that compounds **1** and **2** have affinity to bind the ASP243 residue of NF-κB transcription factor, an amino acid located in the protein–protein interaction area. Therefore, the two complexes and especially 2 display a good potential to specifically target NF-κB. Compound **2** exhibits a systematically higher affinity for NF-κB than compound **1** (Figure 6), where −ΔGb ranges from 5.33 to 6.10 kcal/mol for compound **1** and from 5.17 to 6.52 kcal/mol for compound **2**. Although the difference in binding free energy is only very slight on most of the highest ranked binding modes, NF-κB shows a higher preference for compound **2** which forms a stable hydrogen bond with ASP243 throughout all the ten highest binding modes. Additionally, complex 2 shows a higher affinity than 1 towards a specific DNA sequence, particularly stacking to a cytosine base. The IC_50_ values corresponding to standard cisplatin are closer to those of complex 1 in detriment of 2 (Table 2). Based on the IC_50_ values, complex 2 is less toxic than complex 1. The cytotoxicity of compound **2** in cancer cell lines does not differ significantly versus the normal cells. According to the in-silico analysis, compound **1** has a better binding capacity towards the asparagine amino acid of HSA. Asparagine plays a critical role in the mitochondrial mechanism of tumor cells, the cell cycle, and apoptosis [57] and the compounds cytotoxicity was evaluated relying on the mitochondrial activity of cells (MTS assay, as described in 3.4. Materials and Methods section). Therefore, our results are consistent with previous findings. Complex 2 has a better selectivity towards certain amino acids of HSA as well, which confirms once again that despite the weaker antiproliferative activity, compound **2** it is target-specific towards the DNA, has, and NF-κB transcription factor. Our preliminary results on these palladium(II) complexes bring into consideration their antiproliferative potential and open the road to further examinations, mainly in vitro, as possible metallodrugs with anticancer activity.

## Figures and Tables

**Table 1:** Basic Crystallographic parameters of palladium(II) complexes **1** and **2**.

Data	Complex 1	Complex 2
Empirical formula	C_29_H_37_N_2_O_4_Pd	C_25_H_29_N_2_O_4_Pd
Temperature (K)/Wavelength (Å)	100(1)/1.54186	100(1)/1.54186
Crystal system/space group	monoclinic, *C*2	monoclinic, *P*2_1_/n
Unit cell dimensions a, b, c (Å) β (°)	a = 26.7722(5) b = 10.2502(1) c = 22.3191(4) β = 100.776(1)	a = 15.4449(1) b = 7.4624(1) c = 25.8668(3) β = 98.978(1)
Formula weight/Volume	584.00/6016.82(2) Å^3^	527.90/2944.78(6) Å^3^
Z, Calculated density	8, 1.289 mg/m^3^	4, 1.191 mg/m^3^
Absorption coefficient/F(000)	5.238 mm^−1^/2424	5.301 mm^−1^/1084
Crystal size	0.26 × 0.1 × 0.07 mm	0.15 × 0.11 × 0.02 mm
2Θ range for data collection	6.722 to 143.506°	6.268 to 143.992°
Index ranges	−31 ≤ h ≤ 32 −12 ≤ k ≤ 6 −27 ≤ l ≤ 26	−19 ≤ h ≤ 13 −9 ≤ k ≤ 8 −22 ≤ l ≤ 31
Reflections collected	123,856	113,553
Independent reflections	8235[R_int_ = 0.0388,R_σ_ = 0.0206]	5714[R_int_ = 0.0409,R_σ_ = 0.0123]
Refinement method	Full-matrix least-squares on F^2^	Full-matrix least-squares on F^2^
Data/restraints/parameters	8235/0/665	5714/0/293
Goodness-of-fit on F^2^	1.011	1.063
Final R indices [I > 2σ(I)] *	R1 = 0.0376 wR2 = 0.1014	R1 = 0.0373 wR2 = 0.1225
R indices (all data)	R1 = 0.0459 wR2 = 0.1044	R1 = 0.0409 wR2 = 0.1277
Flack parameter	0.024(14)	-
Largest diff. peak and hole	0.72 and −0.70 e. Å^−3^	1.07 and −1.06 e. Å^−3^

* *R*[*F*^2^ > 2σ(*F*^2^)], *wR*(*F*^2^), *S.*

**Table 2:** IC_50_ values (μmol/L) of tested compounds in comparison with cisplatin in different cell lines after 72 h of incubation. Data are presented as the mean ± SD of three independent measurements performed in triplicate.

Tested Compounds			
Cell Lines	1	2	Cis-Pt
**A549**	42.79 ± 10.31	86.05 ± 9.85	9.50 ± 0.2
**Caco-2**	24.47 ± 5.28	78.57 ± 7.63	15.20 ± 0.3
**HCT**	18.23 ± 4.87	38.57 ± 7.84	15.30 ± 0.5
**HeLa**	17.35 ± 5.54	34.81 ± 9.22	13.10 ± 0.2
**Jurkat**	5.33 ± 0.98	7.69 ± 1.56	16.20 ± 0.6
**MCF-7**	34.13 ± 7.21	94.28 ± 12.87	15.60 ± 0.3
**MDA**	9.61 ± 1.76	50.10 ± 9.67	17.50 ± 0.5
**3T3**	20.32 ± 5.33	46.36 ± 8.75	20.87 ± 0.3

## Data Availability

Crystallographic data are freely available at the Cambridge Crystallographic Data Centre under 2044620-2044621.

## Supplementary Materials

The following are available online. Additional crystallographic (Figure S1: The crystal packing of complex 1 viewed along the (b) axis. Hydrogen atoms of carbons, multi-disordered acetate anions and solvent molecules are omitted for clarity. Figure S2: The crystal packing of complex 2 viewed between the (a, b, c) axis. Hydrogen atoms of carbons, multi-disordered acetate anions and solvent molecules are omitted for clarity. Table S1. Selected bond distances and angles of palladium(II) complexes 1 and 2. Table S2. Hydrogen Bonds and interactions of complex 1 and 2. Figure S3: Intramolecular and intermolecular hydrogen bonds and Van der Waals interactions in complex 1 Figure S4: Intramolecular and intermolecular hydrogen bonds and Van der Waals interactions in complex 2), DFT, molecular docking (Table S3. Cartesian coordinates for the optimized compounds (1) and (2). Table S4 Docking Results) and cytotoxicity (Figure S5 3D representation of cytotoxicity values for palladium(II) complexes 1, 2 and cisplatin in 8 different cell lines) data are available online.

## Sample Availability

Samples of the compounds are available from the authors.
