# Supplementary material for: Evaluation of Antiproliferative Palladium(II) Complexes of Synthetic Bisdemethoxycurcumin towards In Vitro Cytotoxicity and Molecular Docking on DNA Sequence
Source: Molecules. 2021 Jul 20;26(14):4369. doi: 10.3390/molecules26144369 (PMC8306502; doi:10.3390/molecules26144369)

Supplementary Materials

Evaluation of Antiproliferative Palladium(II) Complexes of Synthetic Bisdemethoxycurcumin towards In Vitro Cytotoxicity and Molecular Docking on DNA Sequence

Natalia Miklášová [1,*], Peter Herich [1,2], Juan Carlos Dávila-Becerril [3,4], Joaquín Barroso-Flores [3,4,*], Eva Fischer-Fodor [5], Jindra Valentová [1], Janka Leskovská [1], Jozef Kožíšek [2], Peter Takáˇc [6,7] and Ján Mojžiš [6]

[1] Department of Chemical Theory of Drugs, Faculty of Pharmacy, Comenius University in Bratislava, Kalinčiakova 8, 83104 Bratislava, Slovakia; peter.herich@stuba.sk (P.H.); valentova@fpharm.uniba.sk (J.V.); leskovska5@uniba.sk (J.L.)

[2] Department of Physical Chemistry, Faculty of Chemical and Food Technology, Slovak University of Technology, Radlinského 9, 81237 Bratislava, Slovakia; jozef.kozisek@stuba.sk

[3] Instituto de Química Universidad Nacional Autónoma de México Circuito Exterior s/n Ciudad Universitaria, 04510 Ciudad de México, Mexico; juadb06@gmail.com

[4] Centro Conjunto de Investigación en Química Sustentable UAEM-UNAM, Carretera Toluca-Atlacomulco Km 14.5, C.P. 50200 Toluca Estado de México, Mexico

[5] Tumor Biology Department, Institute of Oncology "Prof. Dr. Ion Chiricut,a", 400015 Cluj-Napoca, Romania; fischer.eva@iocn.ro

[6] Tumor Biology Department, Institute of Oncology "Prof. Dr. Ion Chiricut,a", 400015 Cluj-Napoca, Romania; fischer.eva@iocn.ro

[7] Department of Pharmacology and Toxicology, University of Veterinary Medicine and Pharmacy, Komenského 73, 04181 Košice, Slovakia

* Correspondence: miklasova@fpharm.uniba.sk (N.M.); jbarroso@unam.mx (J.B.-F.); Tel.: +421-250-117-327 (N.M.); +52-722-276-6610 (ext. 7754) (J.B.-F.)

X-Ray structures for palladium(II) complexes **1** and **2**

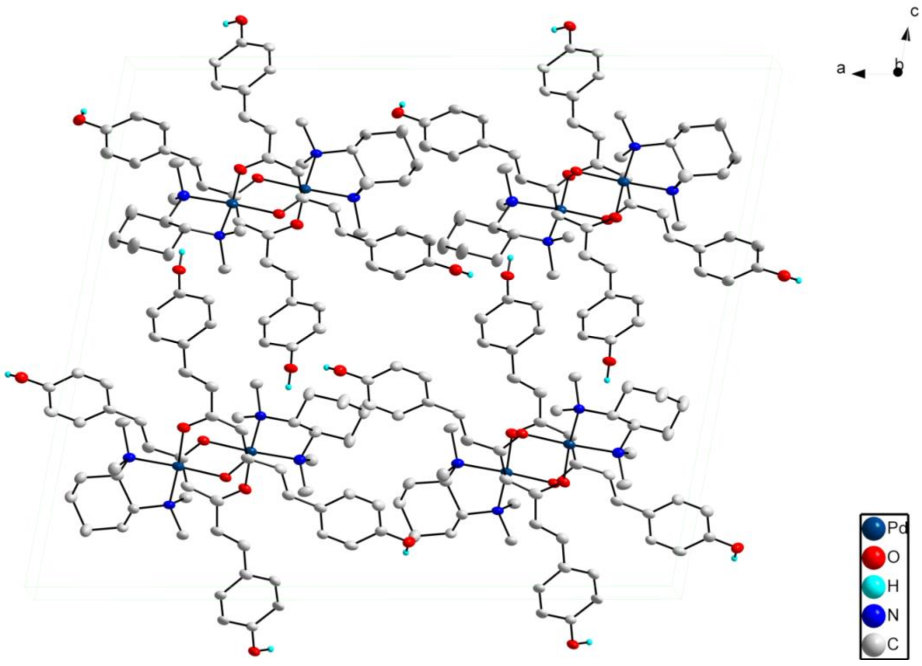

Figure S1: The crystal packing of complex **1** viewed along the (b) axis. Hydrogen atoms of carbons, multi-disordered acetate anions and solvent molecules are omitted for clarity.

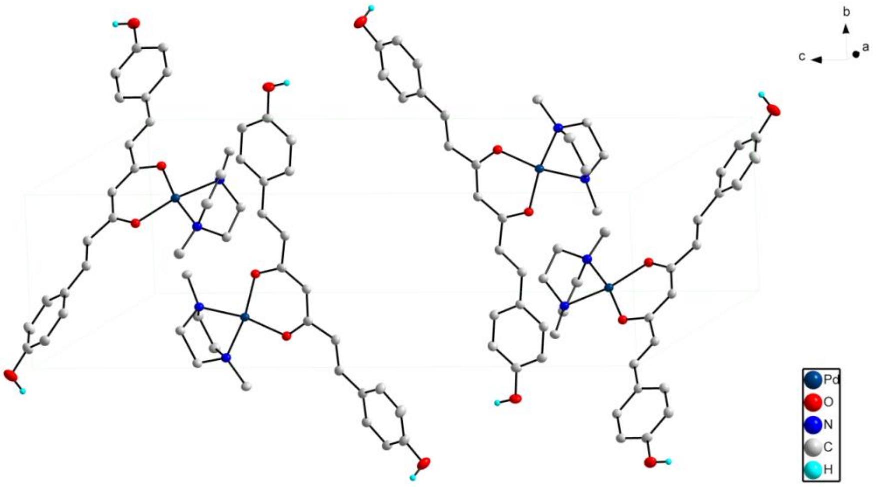

Figure S2: The crystal packing of complex **2** viewed between the (a, b, c) axis. Hydrogen atoms of carbons, multi-disordered acetate anions and solvent molecules are omitted for clarity.

Table S1. Selected bond distances and angles of palladium(II) complexes **1** and **2**.

**Complex 1**				**Complex 1**				**Complex 2**			
**Atom**	**Atom**	**Distance/Å**		**Atom**	**Atom**	**Distance/Å**		**Atom**	**Atom**	**Distance/Å**	
Pd1	N1	2.042(5)		Pd2	N3	2.039(5)		Pd1	N1	2.042(2)	
Pd1	N2	2.057(4)		Pd2	N4	2.051(5)		Pd1	N2	2.036(2)	
Pd1	O1	2.007(4)		Pd2	O5	2.002(4)		Pd1	O1	1.991(2)	
Pd1	O2	1.995(4)		Pd2	O6	1.982(4)		Pd1	O2	1.987(2)	
**Atom**	**Atom**	**Atom**	**Angle/°**	**Atom**	**Atom**	**Atom**	**Angle/°**	**Atom**	**Atom**	**Atom**	**Angle/°**
O1	Pd1	N1	89.2(2)	O5	Pd2	N3	90.3(2)	O1	Pd1	N1	96.41(9)
O1	Pd1	N2	175.3(5)	O5	Pd2	N4	176.6(2)	O1	Pd1	N2	169.63(9)
O2	Pd1	O1	94.7(2)	O6	Pd2	O5	94.7(2)	O2	Pd1	O1	94.99(8)
O2	Pd1	N1	176.0(2)	O6	Pd2	N3	173.5(2)	O2	Pd1	N1	168.37(9)
O2	Pd1	N2	89.8(3)	O6	Pd2	N4	88.5(3)	O2	Pd1	N2	95.36(9)
N1	Pd1	N2	86.3(3)	N3	Pd2	N4	86.6(3)	N1	Pd1	N2	73.22(10)

Table S2. Hydrogen Bonds and interactions of **complex 1** and **2**.

**Complex 1**						
**D**	**H**	**A**	**d(D-H)/Å**	**d(H-A)/Å**	**d(D-A)/Å**	**D-H-A/°**
C2	H2	$O4^{ii}$	0.95	2.44	3.315(7)	153.3
C12	H12	$O4^{ii}$	0.95	2.60	3.409(8)	143.7
C26	H26A	O1	0.98	2.43	3.033(7)	119.0
C27	H27C	O1	0.98	2.52	3.091(11)	116.9
C28	H28A	$O3^{iii}$	0.98	2.61	3.337(9)	130.8
C29	H29A	O2	0.98	2.37	2.914(8)	114.7
O7	H7A	O4	0.84	1.68	2.483(6)	158.2
O8	H8A	$O3^{iv}$	0.84	1.69	2.488(6)	158.5
C41	H41	$O8^{v}$	0.95	2.58	3.375(8)	141.8
C55	H55A	O5	0.98	2.38	2.951(10)	116.1
C55	H55C	$O1^{vi}$	0.98	2.63	3.514(10)	149.6
C57	H57C	O6	0.98	2.50	3.066(9)	116.5
C58	H58A	O6	0.98	2.44	3.001(8)	115.9
C31	H31	$O8^{v}$	0.79(4)	2.62(5)	3.298(8)	144(4)
**Complex 2**						
**D**	**H**	**A**	**d(D-H)/Å**	**d(H-A)/Å**	**d(D-A)/Å**	**D-H-A/°**
C25	H25B	$O4^{i}$	0.98	2.65	3.343(4)	128.3

(i)2-X, 3-Y, 1-Z; (ii)3/2-X,-1/2+Y,1-Z; (iii)-1/2+X,1/2+Y,+Z; (iv)-1+X,+Y,-1+Z; (v)1/2-X,1/2+Y,-Z; (vi)1-X,1+Y,1-Z

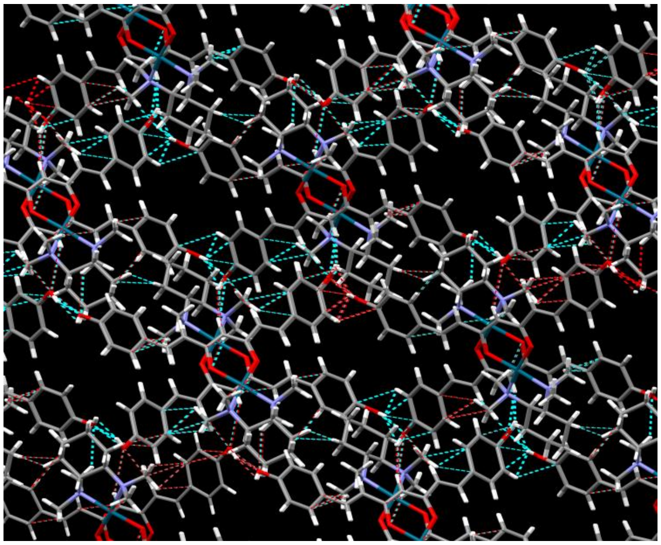

Figure S3: Intramolecular and intermolecular hydrogen bonds and Van der Waals interactions in complex **1**

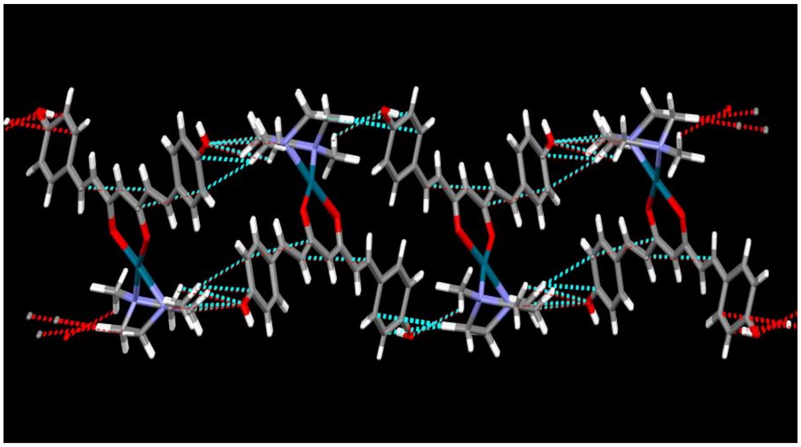

Figure S4: Intramolecular and intermolecular hydrogen bonds and Van der Waals interactions in complex **2**

DFT calculations

Table S3. Cartesian coordinates for the optimized compounds (**1**) and (**2**)

Compound (1)

Pd	-0.00001	1.21340	0.00003
O	-1.46182	-0.16760	-0.00243
O	1.46186	-0.16755	0.00252
O	8.63024	-4.61907	-0.02093
H	9.43148	-4.07133	0.04381
N	1.42745	2.73208	-0.04656
C	-1.26362	-1.47162	-0.00048
N	-1.42754	2.73200	0.04653
C	6.18712	-1.83835	0.11085
H	6.16407	-0.75505	0.19083
C	7.42281	-2.49155	0.10069
H	8.34438	-1.92078	0.17064
C	-3.71502	-1.80942	-0.03587
H	-3.80265	-0.72460	-0.08640
C	0.00005	-2.09564	0.00012
C	7.45748	-3.89209	-0.00160
C	-7.42271	-2.49169	-0.10044
H	-8.34429	-1.92091	-0.17018
O	-8.63010	-4.61925	0.02080
H	-9.43137	-4.07151	-0.04375
C	-6.18703	-1.83847	-0.11057
H	-6.16400	-0.75516	-0.19029
C	3.71511	-1.80931	0.03595
H	3.80272	-0.72449	0.08645
C	1.48070	5.29589	0.00958
H	2.44443	5.29544	-0.51013
H	1.68694	5.31602	1.08644
C	-5.04020	-3.96457	0.07904

H	-4.13066	-4.55080	0.15299
C	2.45923	-2.31976	-0.00430
H	2.28192	-3.38947	-0.04123
C	1.26369	-1.47158	0.00067
C	0.68563	4.02352	-0.34778
H	0.52252	4.01771	-1.43334
C	-6.26198	-4.62885	0.09166
H	-6.32275	-5.70716	0.17193
C	-7.45737	-3.89225	0.00152
C	5.04033	-3.96441	-0.07935
H	4.13080	-4.55063	-0.15357
C	4.97509	-2.55338	0.02299
C	2.43335	2.43781	-1.11801
H	2.85810	1.45114	-0.93111
H	3.22782	3.19300	-1.12018
H	1.93865	2.42393	-2.09098
C	-0.68580	4.02349	0.34774
H	-0.52270	4.01771	1.43331
C	-2.43348	2.43770	1.11794
H	-2.85811	1.45097	0.93107
H	-3.22803	3.19280	1.12001
H	-1.93884	2.42393	2.09094
C	-4.97499	-2.55351	-0.02297
C	-2.45912	-2.31984	0.00448
H	-2.28179	-3.38953	0.04152
C	2.11441	2.71735	1.28585
H	1.41553	2.99311	2.07780
H	2.96513	3.40704	1.29621
H	2.47249	1.70274	1.46869
C	0.68194	6.56158	-0.35513
H	1.25778	7.44712	-0.07104
H	0.53347	6.61245	-1.44190
C	-1.48095	5.29580	-0.00966
H	-1.68721	5.31587	-1.08652
H	-2.44467	5.29532	0.51006
C	6.26211	-4.62868	-0.09202
H	6.32291	-5.70697	-0.17257
C	-2.11444	2.71719	-1.28591
H	-1.41553	2.99298	-2.07783
H	-2.96520	3.40683	-1.29633
H	-2.47243	1.70255	-1.46874
C	-0.68227	6.56156	0.35498
H	-0.53381	6.61251	1.44175
H	-1.25816	7.44704	0.07083
H	0.00007	-3.17861	0.00016

Compound (2)

Pd	0.00000	1.80566	0.00006
O	-1.45592	0.43867	0.00004
O	1.45594	0.43868	0.00024
O	8.75967	-3.73789	-0.00009
H	8.75491	-4.71065	0.00025
N	1.23382	3.49426	0.00005
N	-1.23382	3.49427	-0.00013
O	-8.75968	-3.73788	0.00007
H	-8.75493	-4.71064	-0.00029
C	6.18715	-1.15102	-0.00045
H	6.14036	-0.06554	-0.00083
C	-1.26197	-0.86694	0.00001
C	-0.77629	4.24447	1.21883
H	-1.16570	3.73249	2.09946
H	-1.18555	5.26222	1.19848
C	1.26196	-0.86694	0.00007

C	5.07878	-3.30569	0.00051
H	4.18127	-3.91489	0.00094
C	-4.98561	-1.89678	-0.00005
C	4.98561	-1.89678	0.00003
C	-3.71251	-1.17612	-0.00002
H	-3.77975	-0.08883	0.00004
C	-6.18715	-1.15102	0.00044
H	-6.14035	-0.06554	0.00082
C	-7.43307	-1.77463	0.00048
H	-8.35598	-1.20824	0.00088
C	0.00000	-1.49395	-0.00004
H	-0.00000	-2.57687	-0.00016
C	0.77612	4.24438	1.21901
H	1.18550	5.26208	1.19887
H	1.16527	3.73224	2.09965
C	-2.46351	-1.70546	-0.00001
H	-2.29634	-2.77754	0.00003
C	6.31887	-3.94486	0.00049
H	6.37210	-5.03002	0.00089
C	7.49894	-3.17677	-0.00003
C	2.46351	-1.70546	0.00003
H	2.29635	-2.77754	-0.00003
C	3.71251	-1.17612	0.00003
H	3.77975	-0.08883	0.00003
C	-2.70015	3.25058	-0.00020
H	-2.96387	2.66706	-0.88308
H	-3.24837	4.20076	-0.00020
H	-2.96395	2.66699	0.88261
C	0.77629	4.24433	-1.21899
H	1.16570	3.73226	-2.09957
H	1.18554	5.26209	-1.19875
C	2.70015	3.25057	0.00016
H	2.96387	2.66715	0.88310
H	3.24837	4.20076	0.00005
H	2.96395	2.66689	-0.88260
C	-5.07878	-3.30569	-0.00058
H	-4.18129	-3.91490	-0.00105
C	-0.77611	4.24424	-1.21917
H	-1.18550	5.26195	-1.19917
H	-1.16527	3.73198	-2.09976
C	7.43306	-1.77463	-0.00050
H	8.35598	-1.20824	-0.00088
C	-7.49895	-3.17676	0.00000
C	-6.31888	-3.94486	-0.00055
H	-6.37211	-5.03002	-0.00099

Molecular docking

Table S4 Docking Results:

HSA

	Compound 1 - HSA (4F5S)	Compound 2 - HSA (4F5S)
Mode	-ΔG (kcal/mol)	-ΔG (kcal/mol)
1	7.60	7.43
2	7.07	7.31
3	7.07	7.30
4	7.00	7.15
5	6.99	7.13
6	6.96	6.67
7	6.52	6.58
8	6.32	6.19
9	6.24	6.08
10	5.82	6.00

NF-κB

	Compound 1 - KB (1LE5)	Compound 2 – KB (1LE5)
Mode	-ΔG (kcal/mol)	-ΔG (kcal/mol)
1	6.52	6.52
2	6.39	6.39
3	6.35	6.35
4	5.95	5.95
5	5.85	5.85
6	5.74	5.74
7	5.65	5.65
8	5.54	5.54
9	5.43	5.43
10	5.17	5.17

DNA

	Compound 1 - DNA (2GVR)	Compound 2 - DNA (2GVR)
Mode	-ΔG (kcal/mol)	-ΔG (kcal/mol)
1	13.21	11.74
2	13.20	11.69
3	13.07	11.55
4	13.06	10.99
5	11.99	10.08
6	11.98	9.86
7	11.70	9.68
8	10.70	8.86
9	10.51	8.83
10	10.23	8.70

Cytotoxicity of palladium(II) complexes 1 and 2 was determined by MTS (3-(4,5-dimethylthiazol-2-yl)-5-(3-carboxymethoxyphenyl)-2-(4-sulfophenyl)-2H-tetrazolium) assay

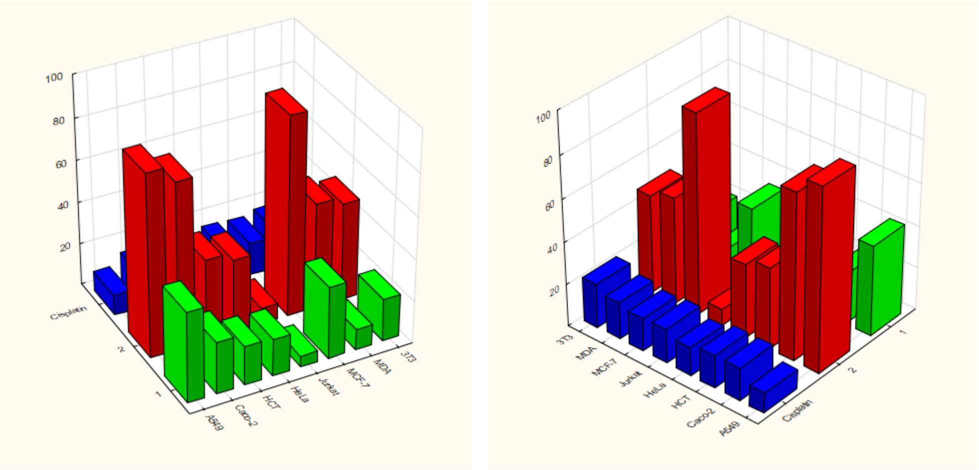

Figure S5 3D representation of cytotoxicity values for palladium(II) complexes **1**, **2** and cisplatin in 8 different cell lines

HRMS, IR and NMR spectra of free ligand and palladium complexes **1** and **2**

HRMS spectrum for **BDMC**

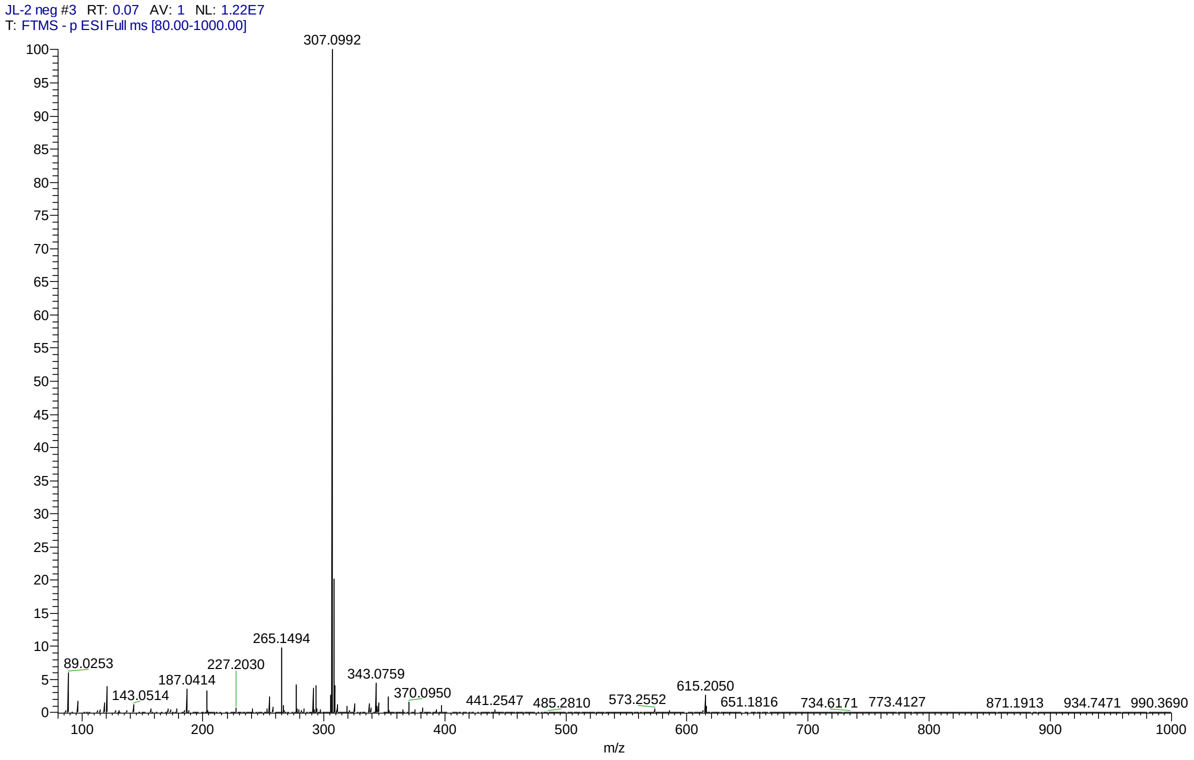

HRMS spectrum of complex **1**

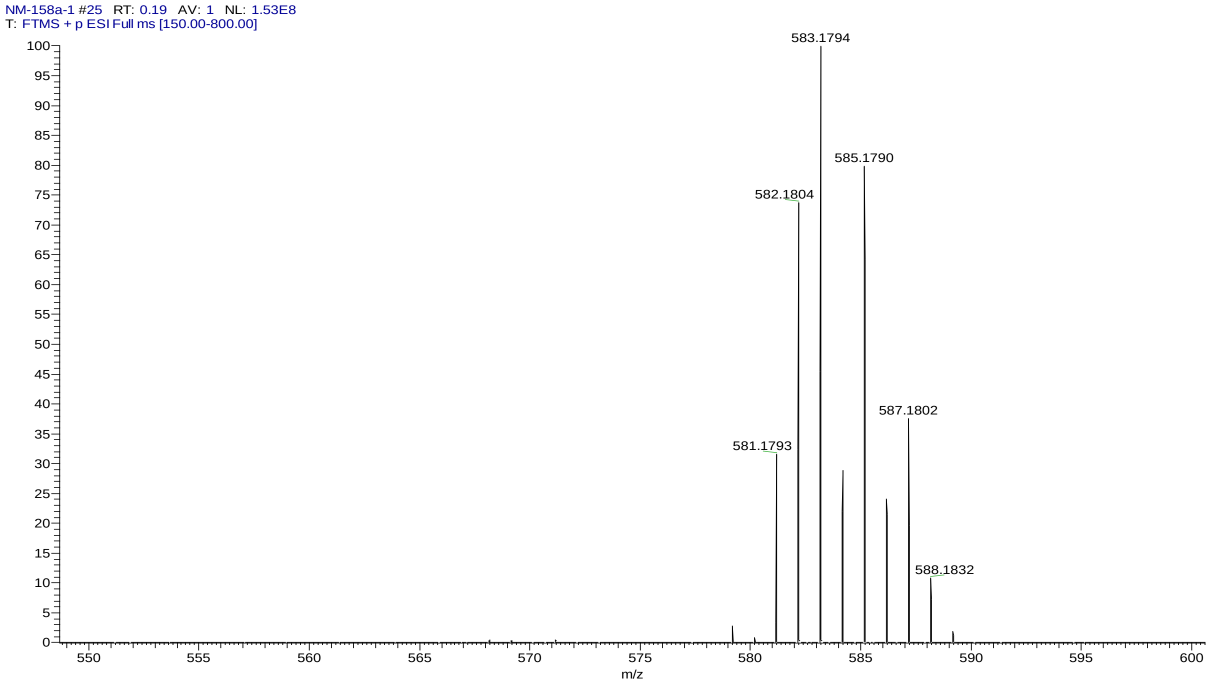

HRMS spectrum of complex **2**

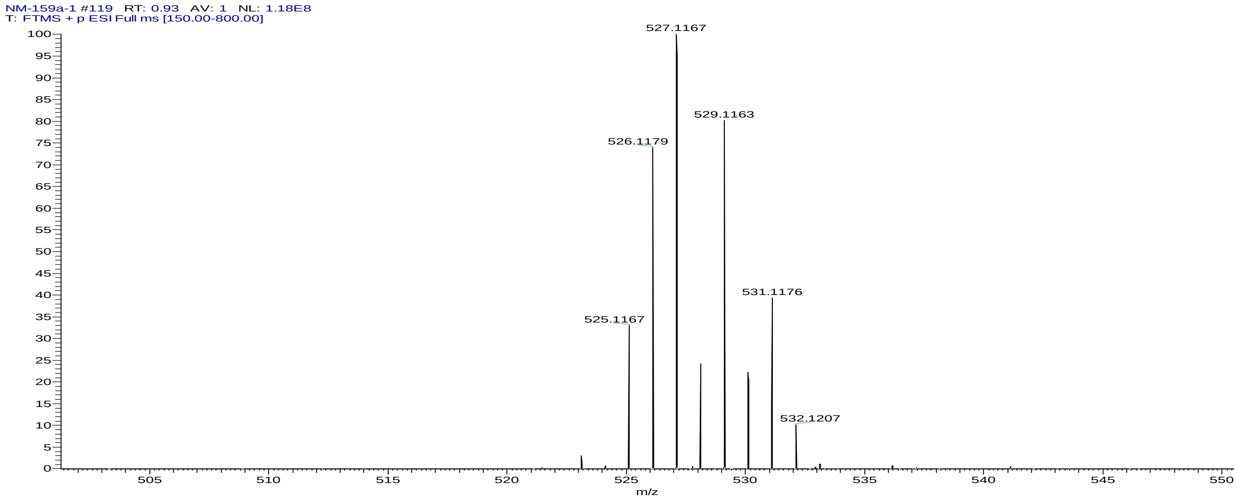

IR spectra for **BDMC**, complex **1** and complex **2**

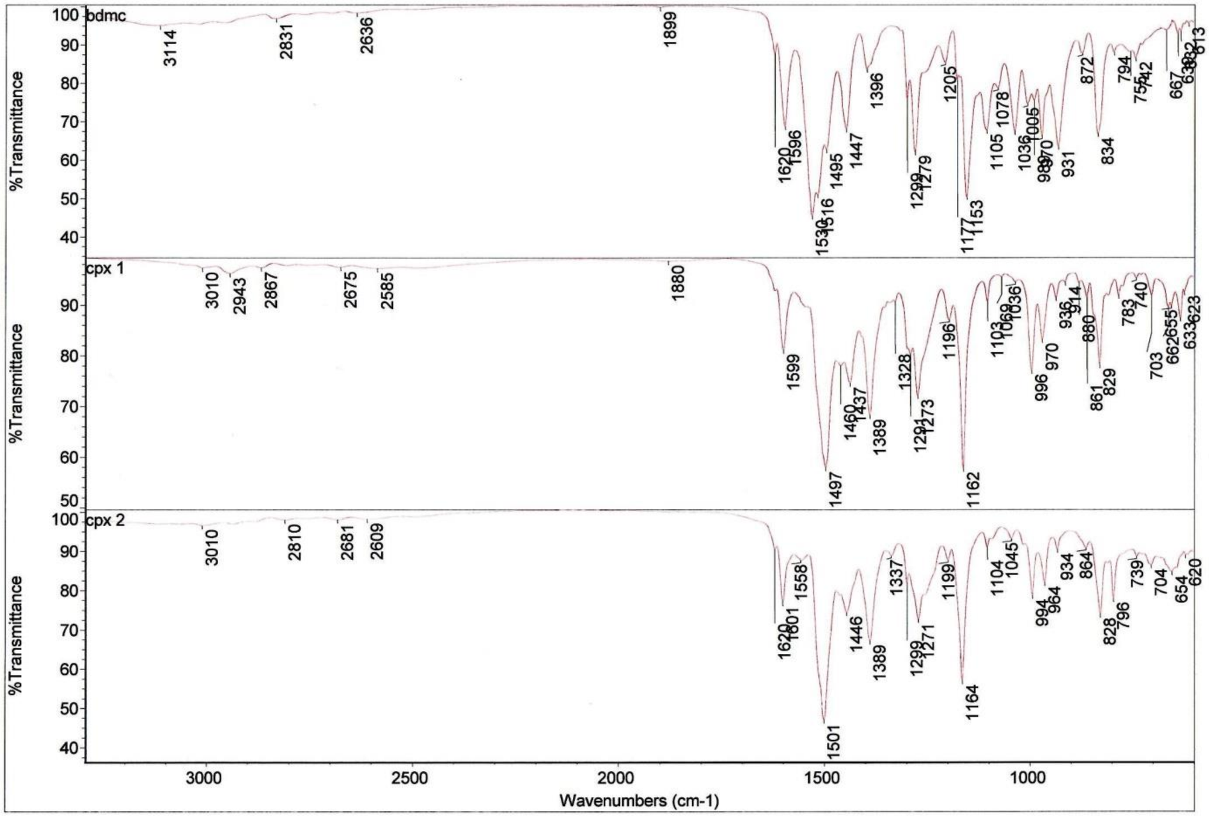

^{1}H-NMR spectrum of **BDMC**

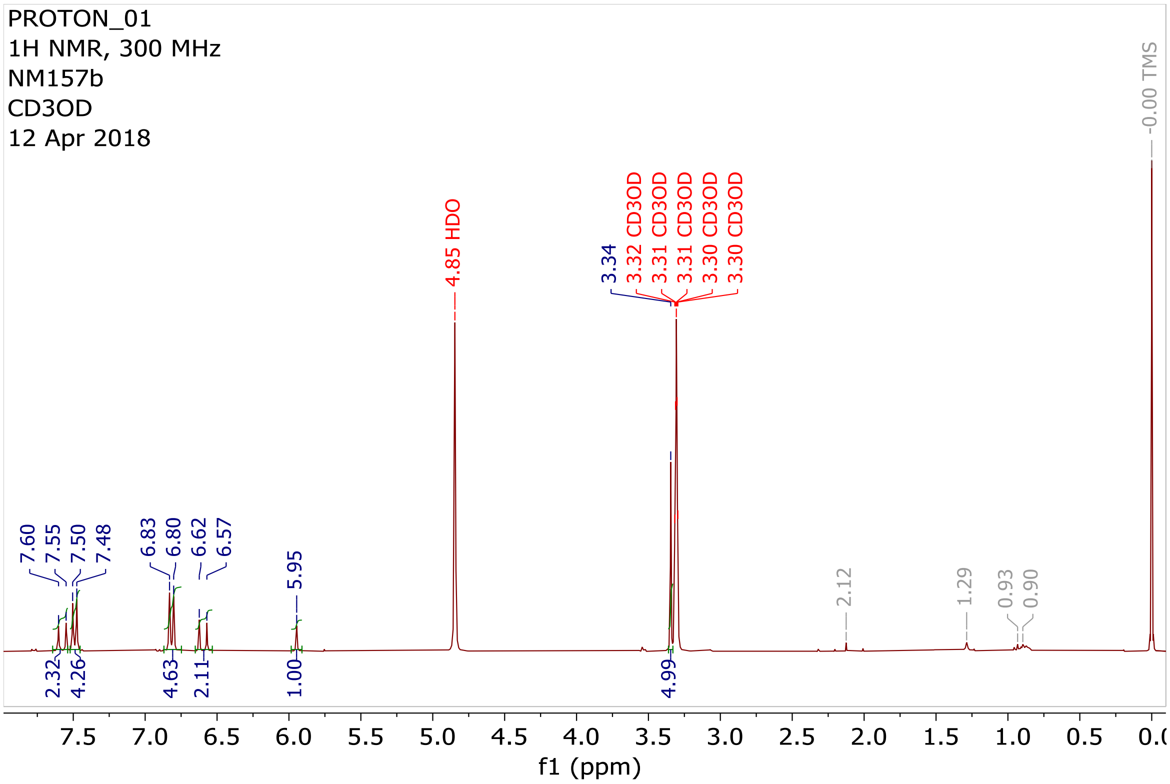

^{13}C-NMR spectrum of **BDMC**

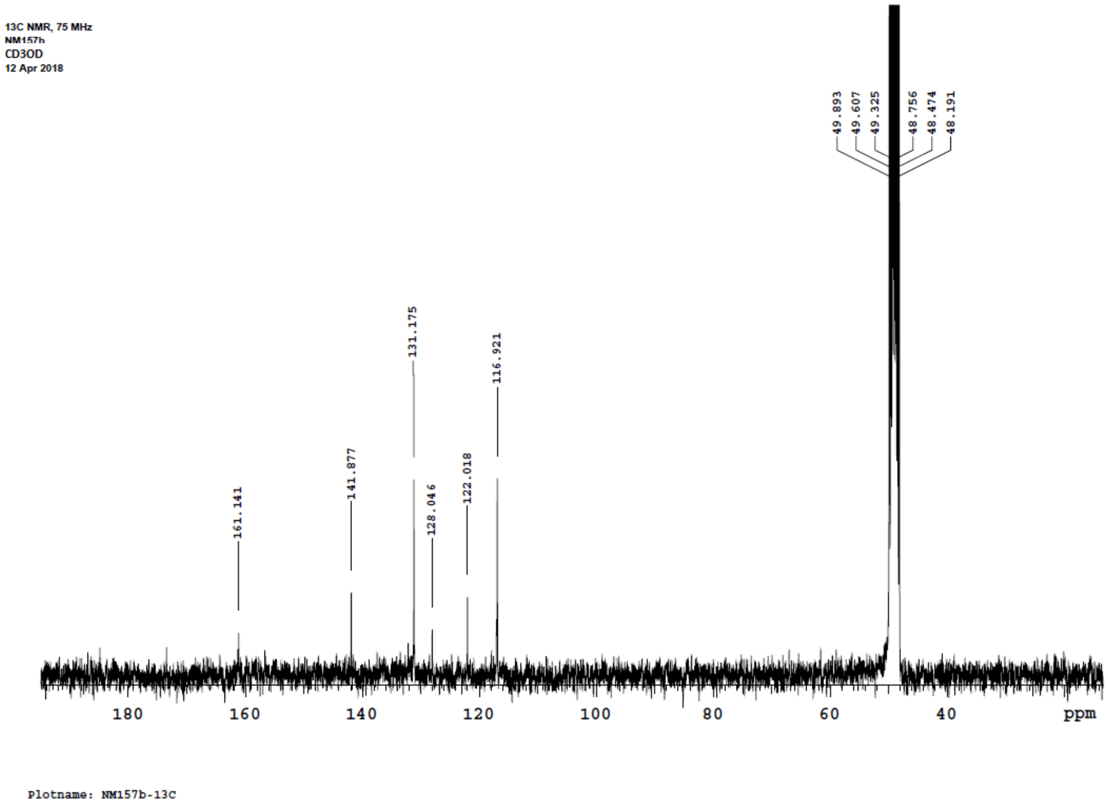

^{1}H-NMR spectrum of palladium intermediate containing *N,N,N',N'*-tetramethylcycohexane-1,2-diamine

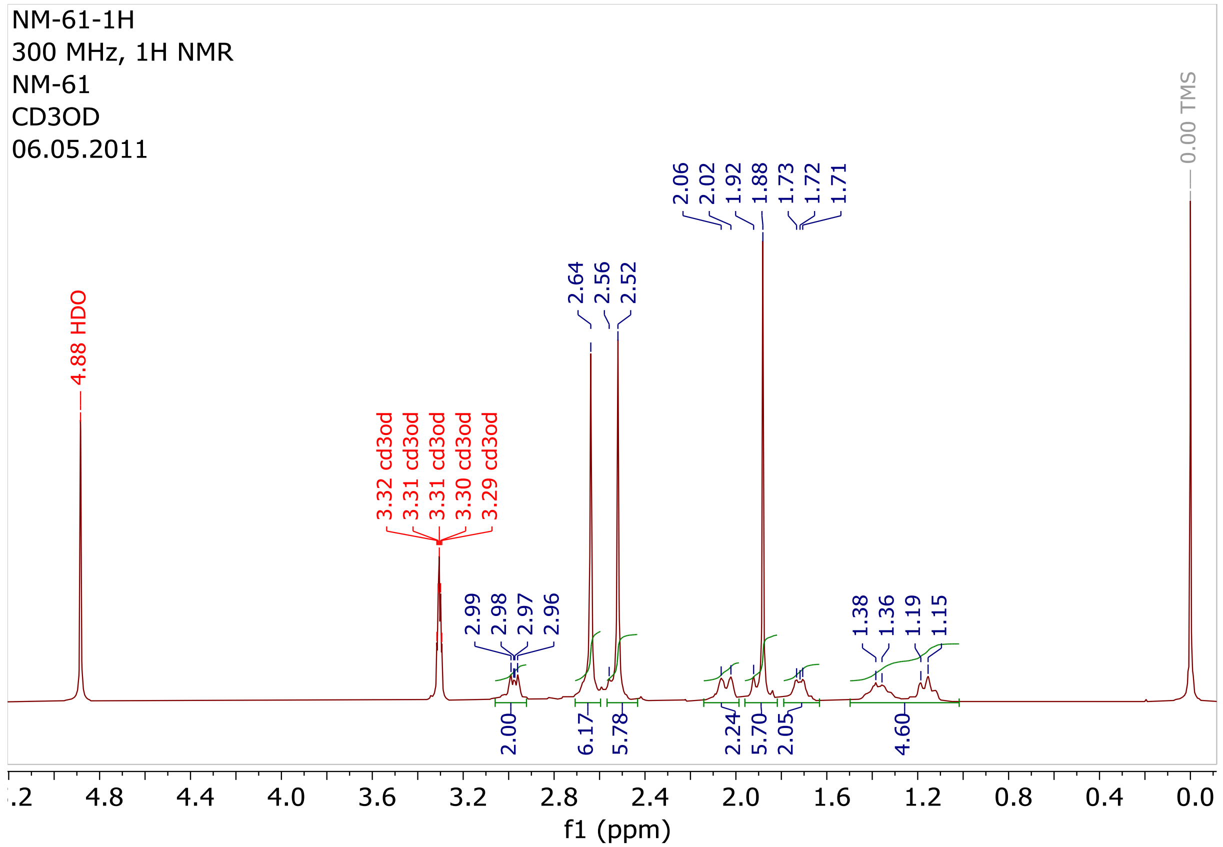

^{13}C-NMR spectrum of palladium intermediate containing *N,N,N',N'*-tetramethylcycohexane-1,2-diamine

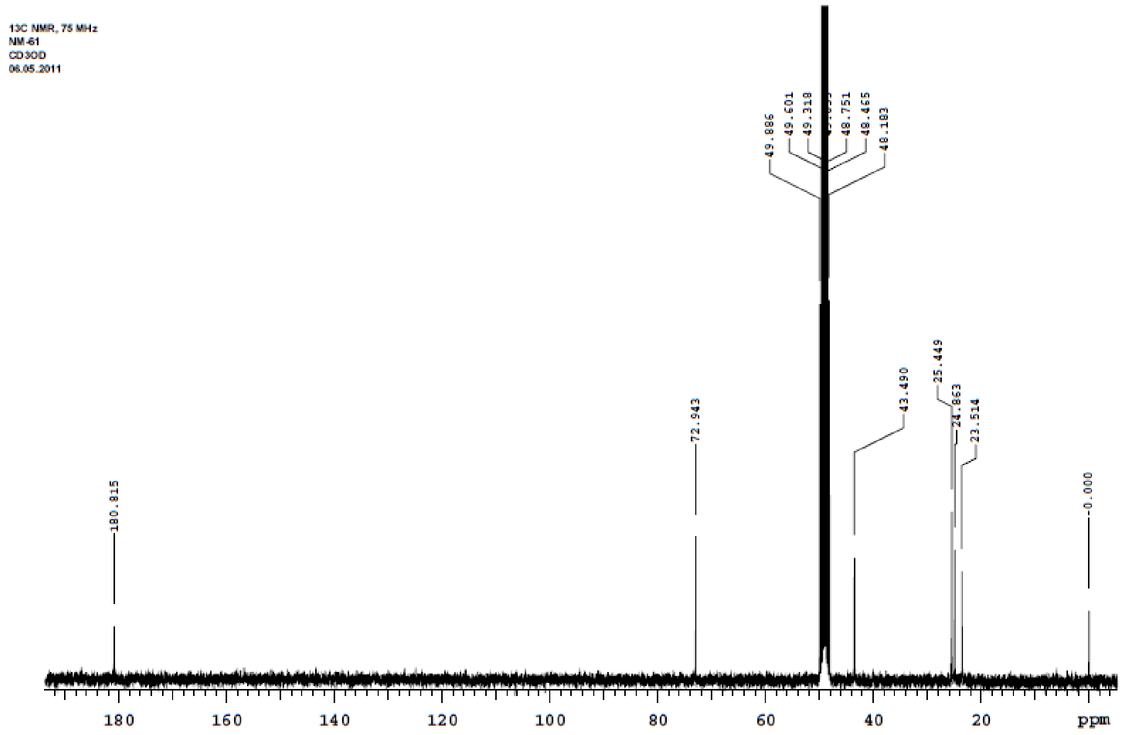

^{1}H-NMR spectrum of palladium intermediate containing *N,N'*-dimethylpiperazine

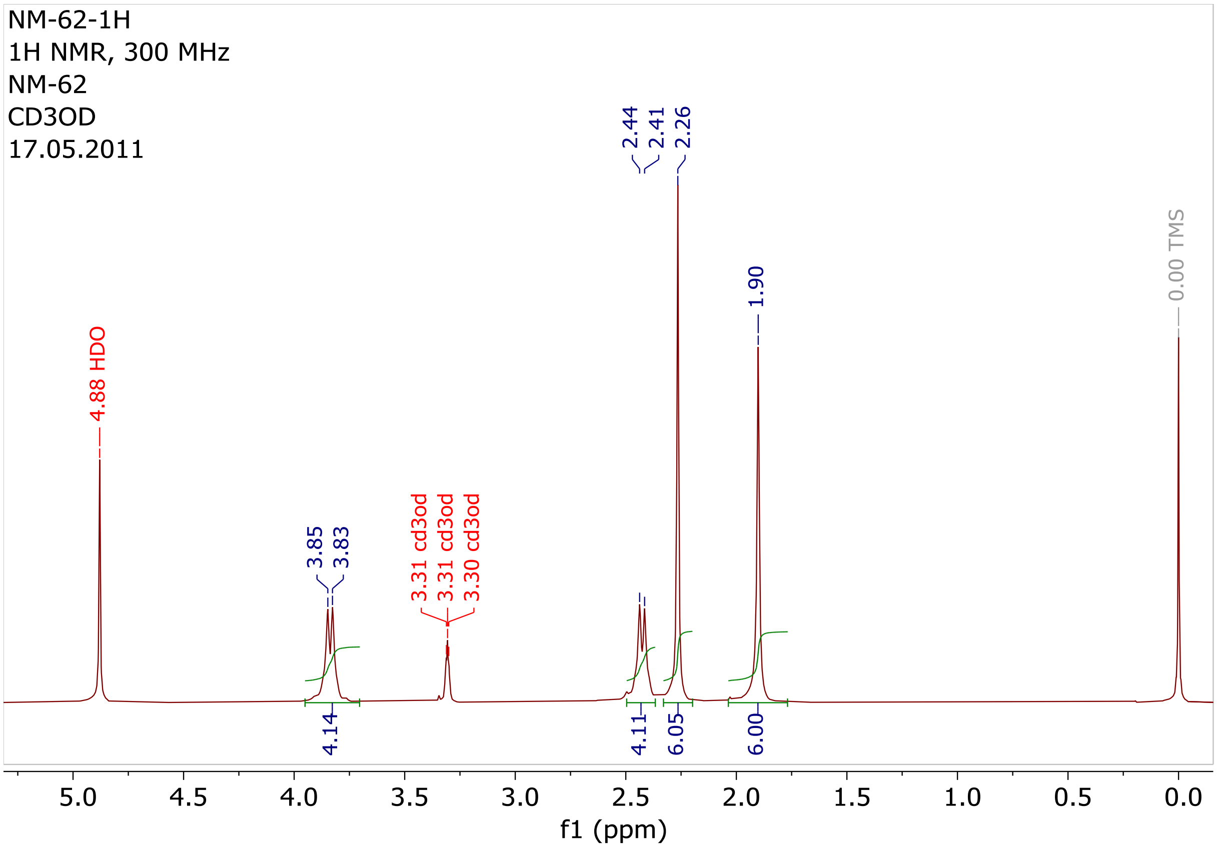

^{13}C-NMR spectrum of palladium intermediate containing *N,N'*-dimethylpiperazine

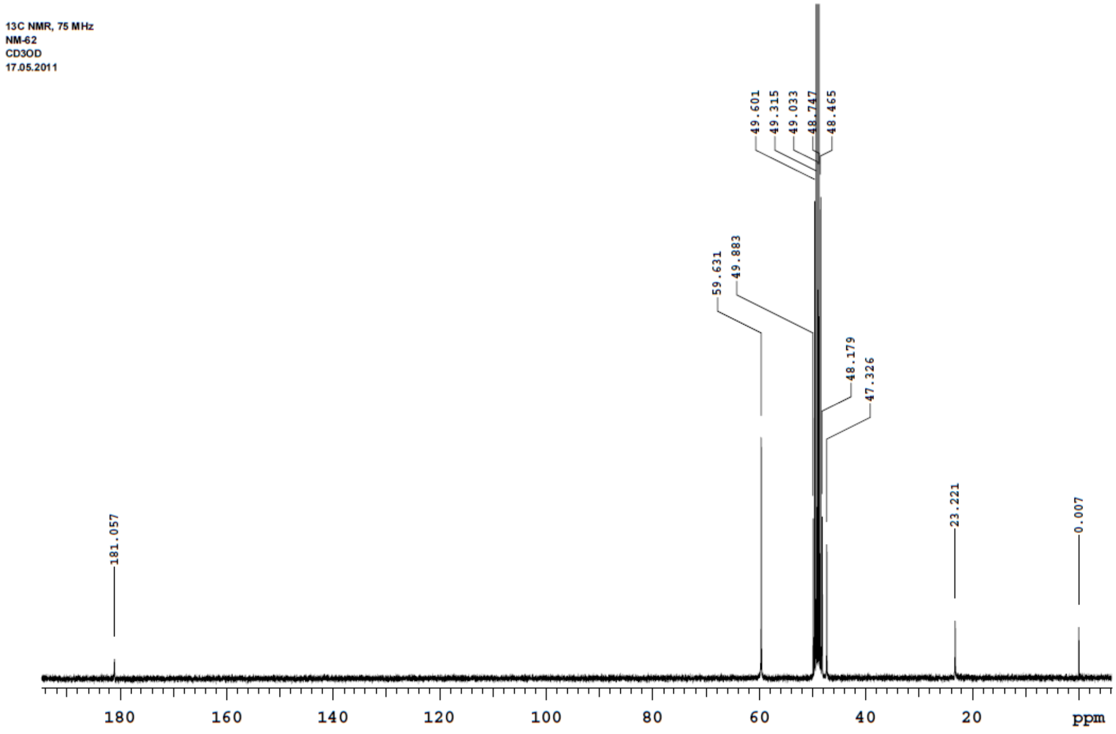

^{1}H-NMR spectrum of palladium complex **1**

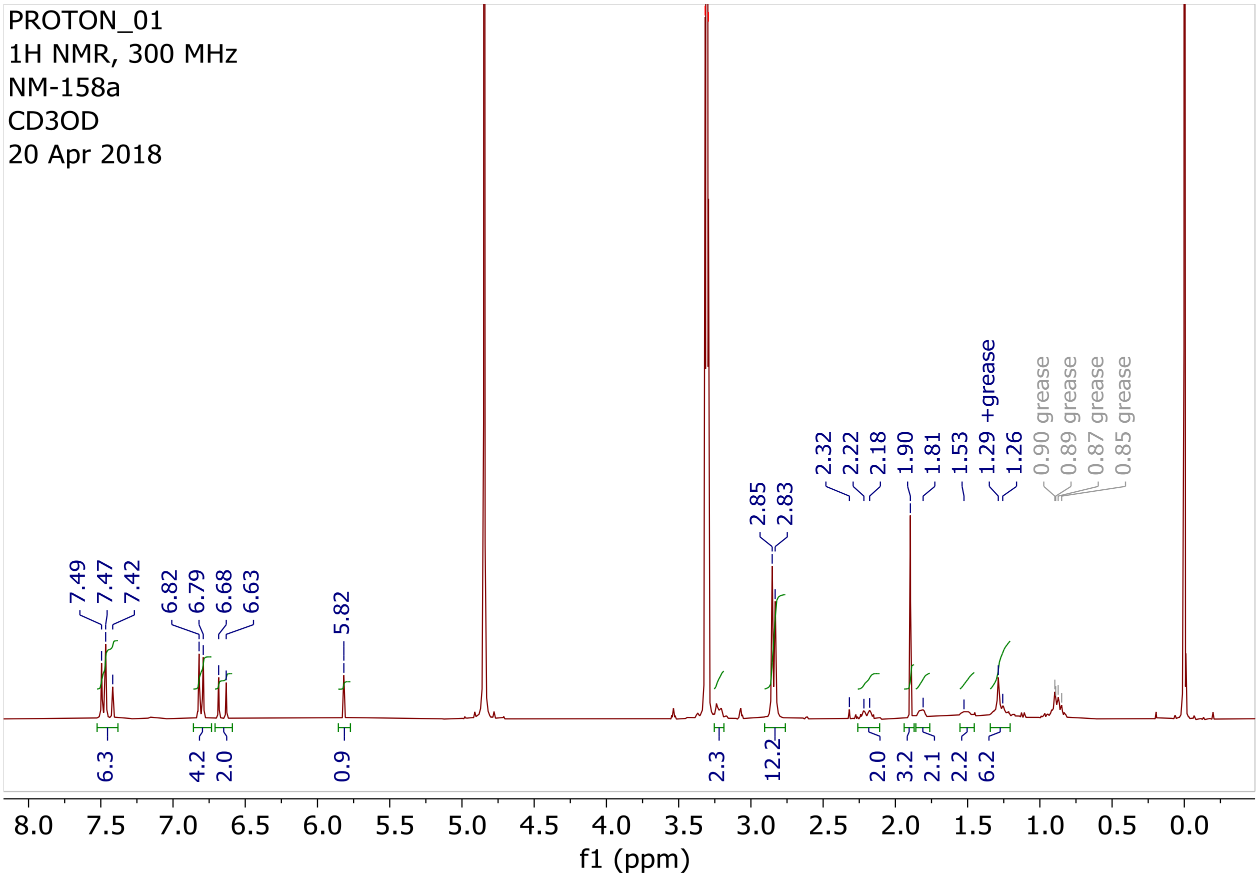

^{13}C-NMR spectrum of palladium complex **1**

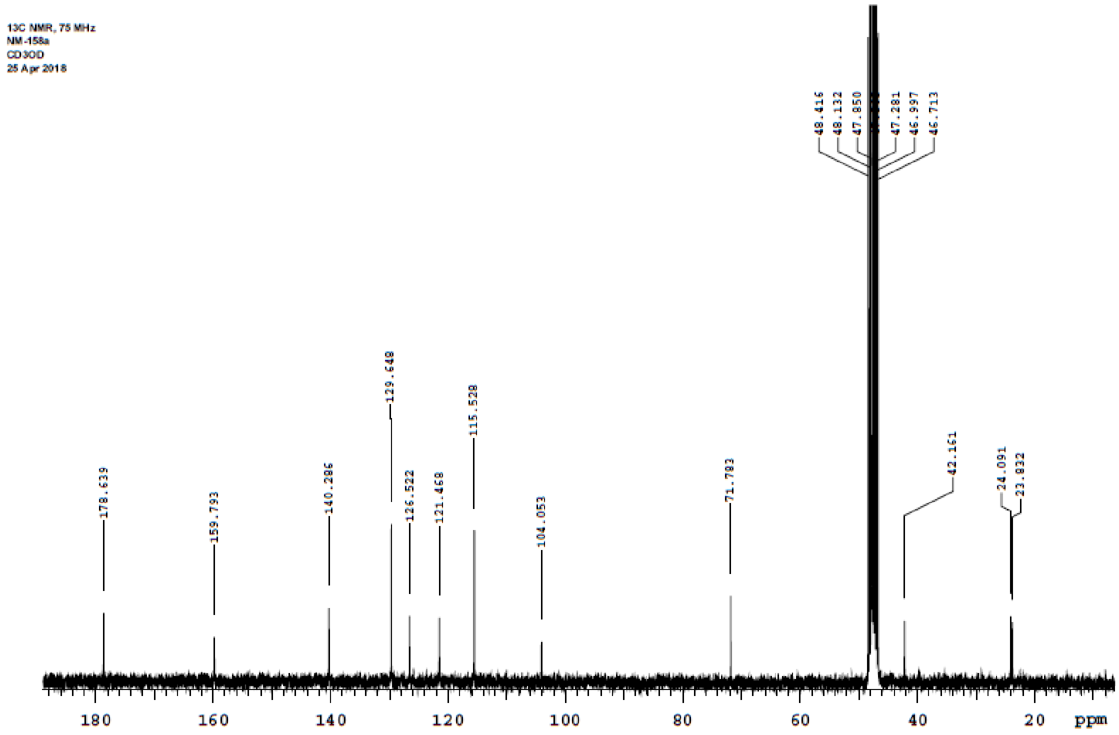

^{1}H-NMR spectrum of palladium complex **2**

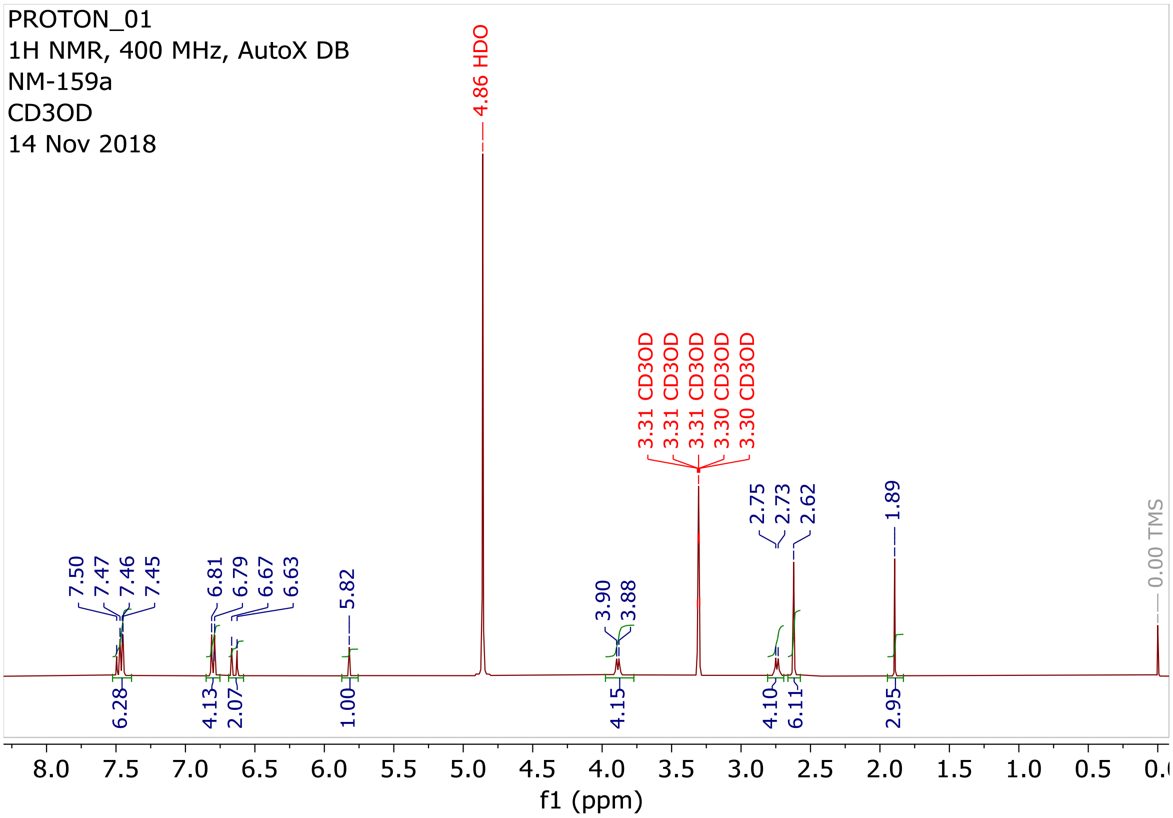

^{13}C-NMR spectrum of palladium complex **2**

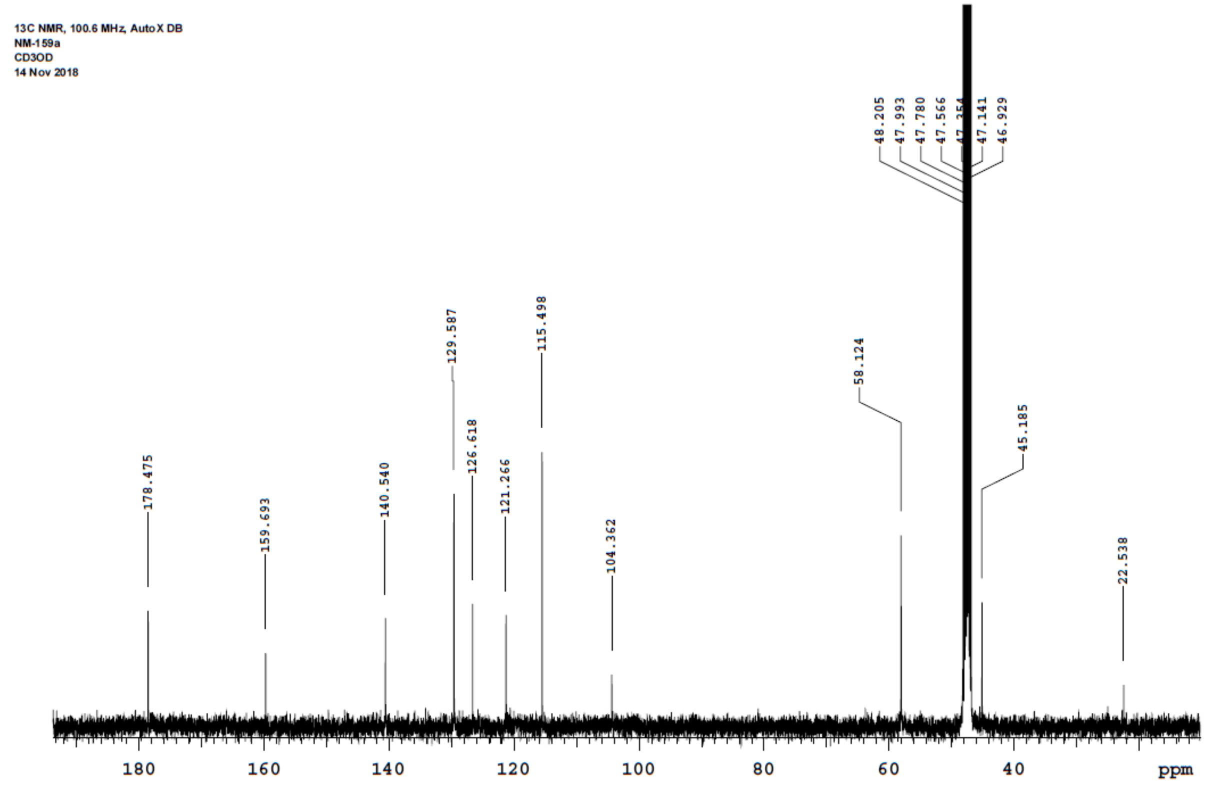

Supplement: Supplementary file 1 [file molecules-26-04369-s001.zip › molecules-1272677-supplementary.pdf]
